# Systematic review of feasibility and acceptability of psychosocial interventions for schizophrenia in low and middle income countries

**DOI:** 10.1186/s12888-015-0400-6

**Published:** 2015-02-12

**Authors:** Carrie Brooke-Sumner, Inge Petersen, Laura Asher, Sumaya Mall, Catherine O Egbe, Crick Lund

**Affiliations:** School of Applied Human Sciences, Discipline of Psychology, University of KwaZulu-Natal, Durban, South Africa; Department of Psychiatry and Mental Health, Alan J Flisher Centre for Public Mental Health, University of Cape Town, Cape Town, South Africa; Department for Population Health, Centre for Global Mental Health, London School of Hygiene and Tropical Medicine, London, UK; Department of Psychiatry, College of Health Science, Addis Ababa University, Addis Ababa, Ethiopia

**Keywords:** Schizophrenia, Community-based, Acceptability, Feasibility, Family intervention, Psychoeducation, Social skills training

## Abstract

**Background:**

In low and middle income countries there is evidence to suggest effectiveness of community-based psychosocial interventions for schizophrenia. Many psychosocial interventions have however been conceptualized in high income countries and assessing their feasibility and acceptability in low and middle income countries is pertinent and the objective of this review.

**Methods:**

Six databases were searched using search terms (i) “Schizophrenia”; (ii) “Low and middle income or developing countries” and (iii) “Psychosocial interventions”. Abstracts identified were extracted to an EndNote Database. Two authors independently reviewed abstracts according to defined inclusion and exclusion criteria. Full papers were accessed of studies meeting these criteria, or for which more information was needed to include or exclude them. Data were extracted from included studies using a predesigned data extraction form. Qualitative synthesis of qualitative and quantitative data was conducted.

**Results:**

14 037 abstracts were identified through searches. 196 full articles were reviewed with 17 articles meeting the inclusion criteria. Little data emerged on feasibility. Barriers to feasibility were noted including low education levels of participants, unavailability of caregivers, and logistical issues such as difficulty in follow up of participants. Evidence of acceptability was noted in high participation rates and levels of satisfaction with interventions.

**Conclusions:**

While there is preliminary evidence to suggest acceptability of community-based psychosocial interventions for schizophrenia in low and middle income countries, evidence for overall feasibility is currently lacking. Well-designed intervention studies incorporating specific measures of acceptability and feasibility are needed.

**Electronic supplementary material:**

The online version of this article (doi:10.1186/s12888-015-0400-6) contains supplementary material, which is available to authorized users.

## Background

Schizophrenia is a chronic and highly disabling mental illness that contributes 15.2 million Disability Adjusted Life Years to the burden of disease in low and middle income countries (LMIC) [1]. Access and adherence to pharmacological treatment is key to improving symptoms and functionality and reducing relapse rates. There is consensus that psychosocial interventions are also an important component of care for schizophrenia. A body of evidence has developed in high income countries (HIC) on five main approaches: psychoeducation [2], family interventions [3], intensive case management [4], cognitive rehabilitation [5] and social skills training [6]. These interventions show reasonable levels of effect on outcomes including relapse prevention, reducing hospital readmission and promoting medication adherence. Within LMIC, community-based rehabilitation, psychoeducation and support for families (delivered by non-specialists) are recommended for low resource settings, with assertive community care and cognitive therapy recommended as additions in higher resourced settings with stronger service-delivery platforms [7]. A recent systematic review of randomized controlled trials for psychosocial interventions for schizophrenia in LMIC suggested evidence for positive effects on social functioning but highlighted a lack of evidence from high quality trials [8]. Studies not included in that review (e.g., non-randomized studies and those with outcomes other than social functioning) also suggest effectiveness of psychosocial interventions in LMIC (for example family interventions in Iran [9], community-based rehabilitation in India [10,11], modified assertive community treatment in South Africa [12], and social skills training in Mexico [13]).

Feasibility as a construct in public health practice incorporates a variety of aspects of intervention delivery. These include demand (is the intervention taken up?), implementation (can it be delivered as planned?), practicality (can it be delivered despite constraints, e.g., of resources and time?) [14]. In addition it incorporates acceptability, or how the recipients of (or those delivering) the intervention perceive and react to it [14]. Assessing the feasibility in LMIC of interventions that have been developed in HIC is particularly pertinent given the variation in available resources and cultural contexts. Furthermore, a critique of some psychosocial interventions has been that their development has been led by service providers, who may lack insight into service users’ perspectives [15]. Information on feasibility, despite being crucial for effective resource allocation, is under-reported in intervention studies [16]. Progress has been made on systematising assessment of feasibility in mental health services in HIC [e.g., Structured Assessment of FEasibility (SAFE)] [16]. However, this approach has yet to be applied extensively in LMIC contexts. The scarcity of resources for mental health services in LMIC, particularly at community level, is well known [17]. The question of feasibility of delivering psychosocial interventions in resource-constrained settings therefore remains. This study aims to systematically assess the evidence for feasibility and acceptability of community-based psychosocial interventions for schizophrenia in LMIC, and to generate recommendations for practice and priorities for future research.

## Methods

Six databases were searched in February 2013 – Medline, Embase, PsychInfo, Global Health, Cumulative Index to Nursing and Allied Health Literature (CINAHL) and the Cochrane Library. Search terms combined three concepts: (i) “Schizophrenia”; (ii) “Low and middle income or developing countries” as defined by World Bank criteria at the time of the study; and (iii) “Psychosocial interventions”. The third concept was expanded in various ways according to the database searched. Interventions captured within this concept included: “Psychotherapy”, “psychoeducation”, “adherence”, “rehabilitation”, “health promotion”, “collaborative care”, “family interventions” and “self-help”. Tailored searches were developed for each of the databases as detailed in Additional file 1, using MeSH terms in Medline and equivalent terms when available in other databases. “acceptability” and “feasibility” were not included as terms in the search strategy. Their inclusion could have reduced the number of abstracts identified, and potentially missed studies that reported on aspects of acceptability and feasibility without specifying this terminology. Community-based intervention for the purposes of this review was defined as an intervention delivered to a person residing in the community rather than in a hospital or other health care facility. The intervention may be delivered at the patient’s home, in a health centre, hospital outpatients’ clinic or other facility. Psychosocial has been defined as an intervention that focuses on psychological, behavioural or social factors, rather than biological factors. For inclusion and exclusion criteria, see Table 1.

**Table 1:** **Inclusion and exclusion criteria**

	**Included**	**Excluded**
**Publication type**	English language	Non-English articles
	Any date	Editorials, review articles, letters, practice guidelines, other guideline documents, conference abstracts, conference reports, news articles
		Grey Literature, Baseline studies
**Study design**	Any study design for primary research that included information relating to the acceptability and/or feasibility of a community-based psychosocial intervention for people with schizophrenia and/or their families and caregivers.	
**Study population**	General adult population.	Interventions for children and adolescents (< 18 years).
	Study conducted in LMIC as defined by the World Bank at time of study.	Study conducted in HIC.
**Condition of interest**	Schizophrenia or schizoaffective disorder only.	Other mental disorders (depression, substance abuse, bipolar disorder, anxiety disorder). Epilepsy, other types of disability. Brief psychotic disorders.
**Intervention**	Any community-based psychosocial intervention delivered to people with schizophrenia or their caregivers.	Pharmacological interventions
		Interventions for hospital in-patients.
**Outcome**	Any quantitative or qualitative measure from service users or care givers showing acceptability and/or feasibility of the psychosocial interventions.	Effectiveness data, when not accompanied by data on acceptability and feasibility.

**Figure 1:**
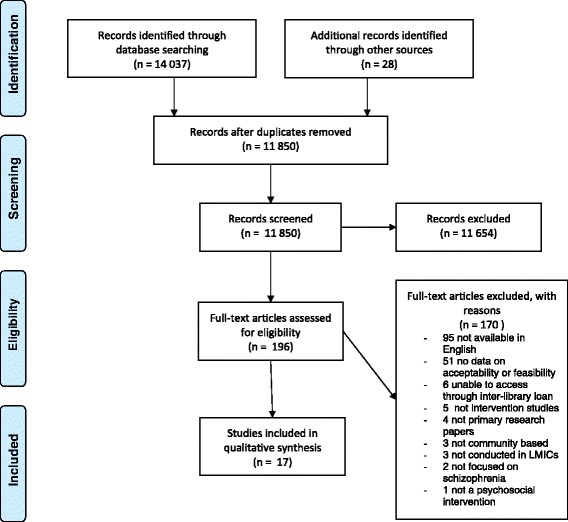
**PRISMA checklist.**

Abstracts identified were extracted to an EndNote Database (14 037 abstracts) (see Figure 1). Relevant researchers were contacted, and reference lists reviewed to identify further studies. CBS and SM independently reviewed the abstracts according to the inclusion and exclusion criteria. Full versions of studies meeting these criteria, or for which more information was needed in order to include or exclude, were accessed (196 full text articles). CBS reviewed these studies in full. SM and COE checked for agreement on included and excluded studies. Data were extracted using a standard form, with data extraction performed by CBS and COE independently (see Additional file 2). These authors then agreed on the final data to be included in the analysis.

Quality of the included studies was assessed by CBS and COE independently using the Effective Public Health Practice Project (EPHPP) Quality Assessment for Quantitative Studies [18], which enables assessment of selection bias, appropriateness of study design, the level of confounding, use of blinding, and the appropriateness of data collection methods and data analysis (see Additional file 3). For qualitative studies the Critical Appraisal Skills Programme (CASP) [19] checklist was used. This tool assesses the appropriateness of the methodology, research design, recruitment strategy, data collection and analysis, and influence of the relationship or bias of the researcher (see Additional file 3). Owing to the small number of studies and limited reporting on acceptability and feasibility, the quality assessment was used to guide understanding of the relative strengths of the evidence rather than to exclude studies [20].

### Data analysis

#### Qualitative synthesis

Data analysis encompassed a qualitative synthesis (thematic synthesis) of qualitative and quantitative data [21]. This method is a three-step process involving (i) free coding of data from included studies; (ii) organisation of free codes into related areas or “descriptive themes” and (iii) inferring “analytical” themes which go beyond the findings of the original studies [21,22]. Studies of weak or unknown quality were not included in the initial analysis but were revisited after analysis of high quality studies to search for additional themes or supporting data. No additional themes emerged, however, supporting data for existing themes were included in this way. Several strategies were adopted to reduce possible bias. Firstly, coding was inductive rather than using an *a priori* framework. Secondly, analytical themes were generated through consensus amongst the authors. In the full article review, nine articles describing seven studies reported anecdotally on feasibility and acceptability. The concepts identified in these anecdotal reports were not operationalised as part of the data collection, however, the data from these studies was used in support of the already identified themes.

## Results

### Characteristics of included studies

Seventeen articles from 17 separate studies were included in the review. The main characteristics and quality assessments for these studies are presented in Table 2. The included studies were from 11 countries in Asia, Africa, South America, the Middle East and Eastern Europe. The studies presented a wide variety of settings, locations and designs as described in Table 2. Interventions in the included studies are described in Table 3.

**Table 2:** **Summary characteristics of included studies**

**Variable**	**Number of studies**	**%**
**Setting**		
Out-patient clinic	11	65%
Community health/rehabilitation centre	3	18%
Home-based	2	12%
Not reported	1	5%
**Location**		
Urban	13	80%
Rural	1	5%
Rural and urban	1	5%
Not reported	2	10%
**Intervention target**		
Individual (patient)	5	30%
Family/caregiver	6	35%
Patient and caregiver	6	35%
**Implementation workforce**		
Lay worker	2	10%
Specialist	12	71%
Not reported	4	19%
**Study design**		
Randomised controlled trial	5	30%
Cohort	3	19%
Cross sectional	4	21%
Qualitative	5	30%
**Quality assessment**		
**Quantitative**		
Adequate	11	85%
Weak/unknown	2	15%
**Qualitative**		
Adequate	4	100%
Weak/unknown	0	0%

**Table 3:** **Description of included studies**

**Country (Author, date)**	**Study design**	**Intervention**	**Intervention workforce**	**Sample**	**Measures of acceptability and feasibility identified**
1. China (Xiong et al., 1994 [40])	Randomised controlled trial (RCT)	1. Monthly 45 minute counselling sessions with patient and family	Therapist	63 families	Compliance
				34 in intervention group	
		2. Family group therapy sessions		29 in control group	
2. China (Ran et al., 2003 [27])	RCT	1. Monthly family visit incorporating psychoeducation	Therapist	326 patients and families	Reasons for refusal to participate
		2. Family workshops		126 cases in family intervention group	
		3. Crisis intervention			
				103 cases in pharmacological treatment group	
				97 cases in control group	
3. Poland (Slupczynska-Kossobudzka et al., 1999 [36])	Cohort	1. Medication management	Multidisciplinary team: psychiatrist, psychologist, 3 nurses, social worker	88 patients and families	Satisfaction scale
		2. Individual psychotherapy			
		3. Daily living and social skills training			
		4. Therapeutic work with family			
		5. Welfare assistance			
4. Turkey (Tas et al., 2012 [35])	Randomised pilot study	1. Family-assisted social cognition and interaction training (14 session group training)	Family members trained as “cognition partners”	45 patients and 45 family members	Satisfaction scale
5. China (Zhang et al., 1993 [49])	Cohort	1. Family psychoeducation (10 lectures, 3 discussion groups)	Psychologist	3092 patients	Participation rates
6. China (Zhang et al., 1994 [25])	Cohort	1. Family counselling	Counsellors	83 patients and family	Description of feasibility issues
		2. Home visits for non-attenders			
7. Egypt, (Gohar et al., 2013 [33])	RCT	1. Social cognition training (2 sessions per week for 8 weeks)	Psychiatrist	42 patients 22 in intervention group	Satisfaction scale
				20 in control group (skills training intervention)	
8. Poland (Chadzynska et al., 2011 [37])	Cross-sectional	1. Group psychoeducation sessions	Therapist	167 patients	Questionnaire covering opinions on sessions
9. Chile (Caqueo-Urízar et al., 2009 [39])	Cross-sectional	1. Multifamily intervention programme for caregivers – 18 weekly sessions (psychoeducation and living skills)	Not reported	41 primary caregivers	Satisfaction questionnaire
10. India (Kulhara et al., 2009 [32])	RCT	1. Manualised psychoeducation intervention for carers (monthly sessions of 1 hr)	Mental health professionals	38 patients and caregivers in both experimental and control groups	Satisfaction questionnaire
11. Brazil (Cabral et al., 2010 [29])	Cross-sectional	1. Weekly psychoeducational and supportive therapy group for patients	Not reported	44 primary caregivers	Opinion questionnaire and satisfaction scale
		2. Weekly psychoeducational multi-family group			
12. Thailand, (Worakul et al., 2007 [34])	Cohort	1. Family psychoeducational programme (1 day programme didactic component and group discussion)	Psychiatrists	91 primary caregivers	Satisfaction scale
13. Czech Republic (Motlova et al., 2006 [38])	Prospective follow up study	1. Outpatient clinic based psychoeducation intervention for patients and family	Professionals (not specified)	53 patients, 93 family members	Outcome questionnaire
14. India (Balaji et al., 2012 [23])	Qualitative	Collaborative community-based care:	Community lay health workers	In-depth interviews with 32 patients, 38 caregivers	Qualitative
		1. Psychoeducation			
		2. Adherence management			
		3. Rehabilitation			
		4. Referral to community agents			
15. Brazil (Zimmer et al., 2006 [24])	Qualitative	1. CBT incorporating cognitive differentiation, social perception, verbal communication, social skills, interpersonal problem solving	Not reported	22 patients’ expressions of perceptions of intervention (written and verbal accounts)	Qualitative
16. South Africa (Pooe et al., 2010 [43])	Qualitative	1. Patient psychoeducation	Not reported	Focus groups with 9 in-patients	Qualitative
				9 out-patients (study did not disaggregate in analysis)	
				Semi–structured interviews with 15 patients	
17. South Africa (Asmal et al., 2013 [41])	Qualitative	1. Family therapy – multi family groups of schizophrenia patients and caregivers	Psychiatric nurse	Semi-structured interviews, 20 patients and 20 family members	Qualitative

This study did not aim to review effectiveness or efficacy of the included interventions. However, it is relevant to report on the effectiveness and/or efficacy data that were present in included studies since an intervention may be acceptable and feasible, but have little or no effect. Of the 17 studies included, 11 included data on efficacy or effectiveness (see Additional file 2). These data indicated positive effects on outcomes such as rehospitalisation rates [23-27], relapse rates [23,26,28], medication adherence [26,28], social functioning [25,29,30], quality of life [29], symptomatology [27,29,31], disability [31] and knowledge of the illness [32,33]. Some studies, however, showed little effect, for example on certain aspects of social functioning [29] and on working status [27]. Similarly, in a study conducted by Worakul *et al.*, small statistically significant improvements in knowledge of the illness, and no improvement in attitude to the illness following a psychoeducation programme, were reported [34].

### Outline of results of data synthesis

The limited data on feasibility available from the studies fell under the theme “barriers to feasibility”. Four themes on acceptability emerged, (i) participants’ satisfaction with the intervention (with a variety of measures used to assess satisfaction); (ii) participation rates; (iii) barriers to acceptability; and (iv) facilitators of acceptability. It was noted in the analysis that themes identified were not distinct. For example, fear of stigma as a barrier to acceptability could also be a barrier to feasibility.

### Barriers to feasibility

#### Education level of participants

Two studies cited low levels of education or literacy as a challenge to feasibility. In an Indian collaborative community-based care programme reported on by Balaji *et al.*, five out of 30 participants could not read, rendering psychoeducational materials inappropriate. However, replacing reading components with verbal explanations was feasible [23]. A Brazilian cognitive behavioural therapy (CBT) programme reported participants having difficulty with writing activities and taking instructions for activities to be done at home [24]. The authors suggested a link between low levels of schooling of participants and low motivation for verbal communication tasks [24].

#### Logistical issues

Three studies reported on logistical challenges to feasibility. Balaji *et al.* in India noted difficulties in the roll-out of their collaborative community-based care programme in that out of 43 patients who consented to be involved only 30 received the intervention because 13 were unreachable or had been hospitalized [23]. Yet the programme itself was considered feasible as there was only one case in which a participant could not afford to travel to an intervention worker to receive care [23]. In a Chinese family counselling programme, five out of 42 patients were lost to follow up due to moving out of the district or leaving the parental home [27]. An Iranian programme involving the training of family members to be case managers for patients with schizophrenia reported anecdotally on concerns for the safety of case managers providing home visits. However, during the year of implementation no dangerous incidents were recorded [26].

#### Availability of caregivers

Five studies reported on the unavailability of caregivers either as part of the results of the study, or anecdotally in the discussion. Balaji *et al.* reported that 25% of caregivers were employed or could not be involved for other reasons [23]. In China, Ran *et al.* reported that of those patients who declined to participate in a programme of home-based psychoeducation and family workshops (8.7%) the majority (77.4%) did so because they had no family member who could participate [27]. In line with these findings a psychosocial intervention for mothers of schizophrenia patients in Iran reported anecdotally that half of eligible mothers refused to participate either because it was inconvenient to attend group sessions, because they were not interested, or because there was no one else who could look after their child while they were away [28]. Similarly, in a Brazilian multi-family group intervention family members who worked were unable to attend as groups took place in the morning. However, for each of the 46 patients, at least one relative attended six or more sessions (total number of sessions not reported) [29]. Anecdotal reporting of a psychoeducation programme for caregivers in Malaysia suggested the lack of participation of female caregivers in the programme could be due to the requirement that they stay at home to care for their family member with schizophrenia [30].

#### Resource constraints

Only two studies related feasibility to resource constraints anecdotally. The first suggested that lack of resources for mental health, particularly for training the required personnel, was a key challenge to the implementation of a social skills training programme in Peru [31]. The second reported that involving non-medical personnel, who were appropriately trained and supervised, reduced the costs of the psychoeducation programme for caregivers in India (estimated cost US$ 25 per family unit) [32], making it feasible in this low resource setting.

### Acceptability

#### Participants’ satisfaction with intervention

Ten studies reported either quantitative or qualitative data on participant satisfaction. These data indicated overall good levels of satisfaction. Two studies (in Turkey and Egypt) of social cognition training showed average satisfaction scores above 8 (10 = excellent) [33,35]. Similarly the Indian study of psychoeducation for carers showed high satisfaction (mean score 11.8, SD 0.8; 12 = highest satisfaction) [32]. Participants in a Thai study of a family psychoeducation programme rated their levels of satisfaction 3 or above out of 5 [34] and a Polish study of a multicomponent intervention (medication management, psychotherapy, social skills training) showed less than 10% dissatisfaction for nine dimensions assessed [36]. In the Brazilian multi-family group therapy intervention the majority of family members found the meetings useful (85%), well organized (75%), and that they helped them to cope with their relative’s illness (99%) [29]. The majority also found the multi-family model an acceptable format [33]. Similarly a Polish study of therapist-delivered group psychoeducation reported that 84% of patients had a positive attitude towards sessions [37]. A Czech study of an outpatient clinic-based psychoeducation intervention for patients and family showed patients acknowledged the importance of the information they gained, the value of sharing experiences and also welcomed relatives being involved [38].

Some studies reported lower levels of satisfaction. In Chile family members in a multifamily psychoeducation and skills building programme showed high levels of satisfaction with the progress of their family member, although overall satisfaction with the service provided was higher in the study control group (a waitlisted group receiving usual care) [39]. The Brazilian CBT study showed good patient satisfaction with training on social perception, social skills and problem solving but low levels of satisfaction with abstract activities on cognitive differentiation and verbal communication [24].

#### Participation rates

Three studies reported participation rates ranging from high to moderate levels. High participation was reported in a Chinese family psychoeducation programme (10 lectures, three discussion groups: 90.3% of participants attended five or more sessions) [27]. A Chinese programme of home-based psychoeducation and family workshops reported an 8.7% refusal rate for those invited to join the programme, and although the refusal rate is a different measure to the participation rate, low levels for the refusal rate suggest high rates of participation [40]. A South African study of multi-family group therapy reported moderate overall participation of 79.5% among relatives and 70.5% among patients [41]. The investigators reported several measures to encourage participation. Sessions were arranged to coincide with scheduled clinic treatment, the study coordinator reminded relatives of sessions by telephone the day before, and participants were reimbursed for their travel costs [41]. A Chinese programme of individual counselling sessions with patients and family group sessions showed similarly moderate rates of participation, with 23% of patients and 27% of families defined as non-compliant (did not attend sessions and refused home visits) [25].

### Barriers to acceptability

#### Fear of stigma

Among family members and patients, fear of stigma linked with the disclosure of diagnosis was reported in four studies. In the Chinese programme of home-based psychoeducation and family workshops, of the 8.7% of people who refused to participate, 22.6% cited fear of social stigma as the reason [27]. Again in China, Xiong *et al.* reported that even with regular contact, 32% of families never attended family group meetings because of fear of discovery of their relative’s illness [40]. Fears of “gossip and ridicule” in the community were common in the collaborative community-based care programme in India [23]. An anecdotal report from another Indian study of home-based cognitive retraining suggested that high dropout rates could be due to families’ fear of stigma associated with attending the psychiatric hospital where the study was located [42].

#### Lack of appreciation of intervention benefits

Balaji *et al.* reported that 24 of 67 families declined to participate as they were “not interested” or thought the intervention would not be helpful [23]. Misunderstanding and suspicions that home visits would be used to try to convert the families to Christianity were voiced. In Brazil, Zimmer *et al.* found that schizophrenia patients could not relate some training exercises to their day-to-day lives and therefore did not fully grasp the benefits of aspects of the CBT intervention [24].

### Facilitators of acceptability

#### Appropriateness of intervention content and materials

Two studies highlighted the importance of appropriate content from the perspective of participants. The Polish group psychoeducation study indicated patients found several psychoeducation topics to be important but difficult to engage with (e.g., coping with symptoms, asking for help, causes of illness) [37]. South African participants in the multi-family group therapy study found the content of sessions to be “relevant and accessible” but patients and relatives were interested in different topics. For example, patients were interested in discussing loneliness and substance abuse, whereas relatives were interested in dealing with their family members’ challenging behaviour [41].

Four studies reported on the appropriateness of materials. Patients and therapists in the Polish group psychoeducation study found illustrations, photos and charts to be most helpful and suggested the use of video and internet resources [37]. Similarly, South African patients assessing the adaptation of psychoeducational materials found the original written materials complicated due to technical language, but said that simplification and using illustrations and examples improved their ease of use [43]. In the Polish study, patients found task books and “tests” to assess their knowledge least acceptable [37]. Similarly, a Mexican study of psychosocial skills training for patients noted anecdotally that inclusion of written tasks and “homework” was highly unacceptable, causing participants to feel anxious [13].

#### Health worker characteristics

Three studies reported on the relevance of personal attributes of those delivering the intervention. Characteristics of health workers were key to improving acceptability of collaborative community care in India. Participants had a preference for female workers, and expected them to be well-trained and knowledgeable on the illness [23]. Anecdotal reports from the same programme showed fluency in local dialects and knowledge of the cultural context to be important [10]. The Polish group psychoeducation study showed being “capable of listening and talking” followed by being “trustworthy”, “effective”, “communicating in a clear and straightforward way”, “patient” and “having extensive knowledge” as the most important characteristics [37].

## Discussion

This study reports on feasibility and acceptability from 17 studies of psychosocial interventions for schizophrenia in 11 LMIC. The small number of included studies stemming from the original search strategies (17 articles from 14 037 abstracts reviewed), speaks to the limited nature of the current evidence base. Implementation of psychosocial interventions is a complex process, embedded in and dependent on the context in which it takes place [16], yet reporting of contextual factors, recognized as key to development of mental health interventions in LMIC [44], was limited in studies in this review. The aim of the review was not to report on efficacy/effectiveness, and studies reporting only on this aspect were excluded. Overall, however, the included studies do suggest important benefits for these interventions on a variety of outcomes, pointing to the relevance of research into factors affecting feasibility and acceptability.

Only one paper (Balaji *et al.*) [23] reported extensively on acceptability and feasibility as operationalised constructs. The level of anecdotal reporting on acceptability and feasibility (nine papers) suggests acknowledgement by investigators of the importance of acceptability and feasibility, however, there remains a lack of operationalisation of these elements in research design. This may reflect a researcher bias towards assessing effectiveness with a lack of attention to patient perspectives and contextual factors [15].

Most studies in this review were based in outpatient clinics, and over 50% were delivered by specialists (Table 1), so despite the positive effects noted, the overall question of the feasibility of these interventions remains for settings with shortages of mental health specialists. The included studies are also overwhelmingly in urban areas. Given recent suggestions that non-specialist delivered psychosocial interventions for schizophrenia may be most suitable as an “initial” service where resources and services are scarce (such as in rural areas) [45], lack of focus on rural populations and non-specialist delivered interventions represents an important gap in the evidence.

Overall, evidence on feasibility identified in this review is limited. A recent study of acceptability and feasibility of task sharing interventions for mental health care found ongoing supportive supervision and adequate training and compensation to be crucial for feasibility [46]. The lack of data identified in this review relating to implementation factors such as training, support, supervision and costing is an important gap and is a challenge in intervention development in this, as in other health areas [16]. All of these implementation factors are directly impacted by the availability of resources (financial, human, and other), well known to be a crucial impediment to the provision of mental health services in LMIC. The lack of reporting on resources required for these psychosocial interventions therefore presents a particular stumbling block to the development of the field in terms of generating evidence of effectiveness of these interventions, to say nothing of scaling up of effective and acceptable interventions to reach populations in need. The report of cost by Kulhara *et al.* [32] is important in this regard as it illustrates the financial feasibility of a psychosocial intervention in a LMIC context. Without more reporting of financial feasibility, the perception that psychosocial interventions for schizophrenia are the realm of specialists, and therefore prohibitively resource-intensive for LMIC settings, may prevail.

Barriers to feasibility emerged in the review, including education levels of participants and availability of caregivers. A significant challenge seems to be maintaining contact with participants over time. This may be particularly relevant for people with schizophrenia who may suffer relapse and be hospitalised, and whose families face multiple stressors including lack of support from other family members, their own illnesses, poverty and lack of access to services.

In relation to acceptability, psychosocial interventions for schizophrenia seem to be generally well accepted by patients and families, indicated by moderate to high levels of participation. However satisfaction (measured with satisfaction scales) and participation rates are open to criticism as markers of acceptability due to the many factors (largely unreported) that may affect satisfaction and participation rates (e.g., desired outcomes, incentives, accessibility of intervention site).

Based on data from the review, the imperative for researchers in the field is operationalisation of feasibility and acceptability as constructs in research designs of pragmatic trials of psychosocial interventions for schizophrenia. The following preliminary recommendations are also made with respect to guiding intervention development to enhance feasibility and acceptability:

### Understanding context

Some elements of psychosocial interventions such as improving empathy of service providers towards service users and providing psychoeducation may be universal. However, other elements, such as expressed emotion may vary amongst cultural groups [47]. This review found variation in participation rates in China for different intervention types (psychotherapy versus psychoeducation), with investigators suggesting this could be explained by the lack of acceptance in this context of “talking therapy” as an effective tool for improving schizophrenia [40]. This illustrates how detailed understanding using qualitative methodologies of participants’ perspectives, needs and desired outcomes, as well as the social environment, is vital [44,48]. This review found personal characteristics of those delivering the intervention to be a driver of acceptability. This aligns well with the recent study of acceptability and feasibility of task sharing for mental health in five countries indicating that understanding the socio-cultural context is essential for identifying appropriate health or other workers to deliver the intervention [46].

### Involving caregivers

Constraints around the involvement of family members were a key barrier to participation in the studies in this review. A detailed and context-specific consideration of how best to engage families (as well as patients) should be a core component of intervention development.

### Consideration of stigma and discrimination

In this review, fear of stigma was found to be a disincentive to participation. Participating in an intervention identifies a person or family as “mentally ill” and seems to discourage participation. In addition to the societal level work needed to address stigma, those trained to deliver psychosocial interventions need particular guidance in supporting participants to deal with experiences of stigma and discrimination and to minimise the potential of the intervention to increase stigma (e.g., by involuntary disclosure).

### Use of appropriate materials

This review highlighted that complex written materials or activities that give a sense of testing knowledge discourage participants and reduce acceptability. This indicates the need to adapt interventions taking into consideration education levels and lived experiences. In low resource settings, an intervention workforce should be trained on how to make the content of material accessible to those who are not able to read or write.

### Systems for maintaining contact with participants

In addition to the difficulties for follow up introduced by hospitalization or relapse of participants, difficulties with tracing participants may be particularly relevant in LMIC with high levels of mobility amongst communities. Feasibility may be improved by incorporation of an effective system for following up participants should they be hospitalised or move to a different area.

### Limitations

There are several limitations to this review due to the developing status of this field of research. Many of the included studies had limited information on how interventions were implemented as this has not yet become the norm for reporting these types of studies. A publication bias may exist and studies showing no or negative effects may have important data on acceptability and feasibility. In addition, the small numbers of participants in many of the included studies may limit the generalisability of findings. Limitations in the process of conducting the review include the exclusion of 95 non-English language articles. A similar review of these non-English language studies would add to these findings, particularly given the dependence of acceptability on cultural factors. The review did not cover effectiveness or efficacy of psychosocial interventions for schizophrenia, and a separate review on this topic is needed. The review also did not report on acceptability and feasibility from the perspective of service providers.

### Future research

While the evidence in LMIC for effectiveness of psychosocial interventions for schizophrenia is growing, pragmatic trials are needed of appropriately adapted interventions that focus not only on effectiveness, but also on feasibility and acceptability. Failure to take into account factors impacting on feasibility and acceptability threatens long-term sustainability and disregards the perspectives of patients and their families. Future studies will benefit from in-depth qualitative intervention development work and piloting, and qualitative evaluation to help understand quantitative findings and elucidate barriers to acceptability and feasibility. Future studies should assess participant satisfaction using specifically designed measures based on participants’ desired outcomes (e.g., employment, social activity, and fulfillment of responsibilities).

## Conclusion

While there is preliminary and limited evidence to suggest acceptability of community-based psychosocial interventions for schizophrenia in LMIC, the evidence for overall feasibility is limited. Important barriers to acceptability and feasibility are the fear of stigma associated with being identified as having a mental illness, or having a family member with mental illness, as well as multiple roles and responsibilities of caregivers making it difficult to engage them in interventions. The field urgently needs well-designed intervention studies incorporating measures of acceptability and feasibility, as well as development of instruments to measure acceptability and feasibility in diverse cultural settings in LMIC.

## Additional files

Additional file 1:
**Search strategy.**
Additional file 2:
**Data extraction table.**
Additional file 3:
**Quality assessment.**
