# Supplementary material for: Systematic review of feasibility and acceptability of psychosocial interventions for schizophrenia in low and middle income countries
Source: BMC Psychiatry. 2015 Feb 12;15:19. doi: 10.1186/s12888-015-0400-6 (PMC4382830; doi:10.1186/s12888-015-0400-6)
Supplement: Additional file 1: — Search strategy. [file 12888_2015_400_MOESM1_ESM.docx]

**Appendix 1**

**Search Strategy**

1. **MEDLINE**

**Schizophrenia**

1. Psychotic disorders/
2. Exp Schizophrenia/
3. Exp Psychotic affective disorders/
4. (Schizophreni* or psychosis or psychoses or psychotic or schizoaffective or Schizophreniform or bipolar disorder or manic depress*).tw
5. Or/1-4

**Psychotherapy**

1. exp Psychotherapy/
2. exp Counseling/
3. Schizophrenic psychology/
4. (Psychotherapy or counselling or counseling or therapy or interpersonal therapy or cognitive behavio?ral therapy or art therapy or music therapy or brief intervention or cognitive retraining or cognitive rehabilitation).tw

**Psycho-education**

1. Health Education/
2. Patient Education as Topic/
3. (psychoeducation* or psycho-education*).tw
4. (Patient* or caregiver* or care-giver* or carer* or family or families) adj3 (education or advice or information or training or support or intervention or livelihood).tw

**Family**

1. Caregivers/
2. Family relations/
3. ((family or families or carer or caregiver or care-giver) adj3 burden).tw

**Adherence**

1. exp Patient Compliance/
2. patient adj3 (compliance or concordance or adherence).tw
3. (antipsychotic* or anti-psychotic* or medication*) adj3 (compliance or concordance or adherence or non-compliance or non-concordance or non-adherence ).tw
4. (Adherence or compliance or medication) adj3 (support or therapy or education or training or advice or information or intervention).tw

**Rehabilitation**

1. exp Rehabilitation/ or Rehabilitation Nursing/ or exp Rehabilitation Centers/
2. Social Adjustment/
3. Cooperative behaviour/
4. Interpersonal relations/
5. Social inclusion.tw
6. (community based rehabilitation or community-based rehabilitation or CBR).tw
7. (rehabilitat* adj3 (home based or home-based or communit*)).tw
8. (Communit* adj3 (vocational training or apprenticeship* or employment placement service* or support network* or self-employ* or supported employ* or social service* or social work*)).tw
9. (Communit* adj3 (personal assistance or personal assistant* or individual support* or disabled people* organi?ation*)).tw
10. (Communit* adj3 (empower* or awareness campaign* or self-advocacy or self-help group* or support group* or women* group* or development group*)).tw
11. (Communit* adj3 inclusi* adj3 (health or education or hous* or social or justice or empower*)).tw
12. Rehabilitation adj3 (vocational or social or personal).tw
13. Training adj3 (Life skill* or social skill* or personal skill* or interpersonal skill* or interpersonal).tw
14. Psychosocial or psycho-social or social or psychological or psychiatric or PSR or vocational or occupational adj3 (intervention* or support or rehabilitation).tw
15. Sustainable livelihood* or livelihood* adj3 (intervention or support).tw
16. Recovery or recovery model or recovery approach or social recovery.tw

**Health promotion**

1. Health promotion/
2. Health adj3 (promotion or advice or information or training or support).tw

**Support group**

1. social support/
2. Self-Help Groups/
3. (Social or peer* or peer-led or peer led or self help or self-help or community or cooperative or co-operative) adj3 (group or support or support group).tw

**Collaborative/ primary/ community-based care**

1. care adj3 (Collaborative or community or community-based).tw
2. (outreach adj3 (service* or care or intervention* or program*)).tw
3. Exp Primary Health Care/
4. Community health services/
5. Home Care Services/
6. Home nursing/
7. Community health nursing/
8. Community networks/ or community mental health services/
9. patient care team/
10. nursing, team/
11. exp social work/
12. community health centers/
13. community mental health centers/
14. community health workers/
15. Outpatients/
16. Ambulatory care facilities/ or outpatient clinics, hospital/
17. Ambulatory care/
18. allied health personnel/
19. nurses aides/
20. psychiatric aides/
21. (nonspecialist* or non-specialist* or allied health or nurse led or community health or village health adj3 (worker* or personnel or team*)).tw
22. or/6-62

**Low and middle income countries**

1. developing countries/
2. (Africa or Asia or Caribbean or West Indies or South America or Latin America or Central America).hw,kf,ti,ab,cp
3. (Afghanistan or Albania or Algeria or Angola or Antigua or Barbuda or Argentina or Armenia or Armenian or Aruba or Azerbaijan or Bahrain or Bangladesh or Barbados or Benin or Byelarus or Byelorussian or Belarus or Belorussian or Belorussia or Belize or Bhutan or Bolivia or Bosnia or Herzegovina or Hercegovina or Botswana or Brazil or Bulgaria or Burkina Faso or Burkina Fasso or Upper Volta or Burundi or Urundi or Cambodia or Khmer Republic or Kampuchea or Cameroon or Cameroons or Cameron or Camerons or Cape Verde or Central African Republic or Chad or Chile or China or Colombia or Comoros or Comoro Islands or Comores or Mayotte or Congo or Zaire or Costa Rica or Cote Ivoire or Ivory Coast or Croatia or Cuba or Cyprus or Czechoslovakia or Czech Republic or Slovakia or Slovak Republic or Djibouti or French Somaliland or Dominica or Dominican Republic or East Timor or East Timur or Timor Leste or Ecuador or Egypt or United Arab Republic or El Salvador or Eritrea or Estonia or Ethiopia or Fiji or Gabon or Gabonese Republic or Gambia or Gaza or Georgia Republic or Georgian Republic or Ghana or Gold Coast or Greece or Grenada or Guatemala or Guinea or Guam or Guiana or Guyana or Haiti or Honduras or Hungary or India or Maldives or Indonesia or Iran or Iraq or Isle of Man or Jamaica or Jordan or Kazakhstan or Kazakh or Kenya or Kiribati or Korea or Kosovo or Kyrgyzstan or Kirghizia or Kyrgyz Republic or Kirghiz or Kirgizstan or Lao PDR or Laos or Latvia or Lebanon or Lesotho or Basutoland or Liberia or Libya or Lithuania or Macedonia or Madagascar or Malagasy Republic or Malaysia or Malaya or Malay or Sabah or Sarawak or Malawi or Nyasaland or Mali or Malta or Marshall Islands or Mauritania or Mauritius or Agalega Islands or Mexico or Micronesia or Middle East or Moldova or Moldovia or Moldovian or Mongolia or Montenegro or Morocco or Ifni or Mozambique or Myanmar or Myanma or Burma or Namibia or Nepal or Netherlands Antilles or New Caledonia or Nicaragua or Niger or Nigeria or Northern Mariana Islands or Oman or Muscat or Pakistan or Palau or Palestine or Panama or Paraguay or Peru or Philippines or Philipines or Phillipines or Phillippines or Poland or Portugal or Puerto Rico or Romania or Rumania or Roumania or Russia or Russian or Rwanda or Ruanda or Saint Kitts or St Kitts or Nevis or Saint Lucia or St Lucia or Saint Vincent or St Vincent or Grenadines or Samoa or Samoan Islands or Navigator Island or Navigator Islands or Sao Tome or Saudi Arabia or Senegal or Serbia or Montenegro or Seychelles or Sierra Leone or Slovenia or Sri Lanka or Ceylon or Solomon Islands or Somalia or Sudan or Suriname or Surinam or Swaziland or Syria or Tajikistan or Tadzhikistan or Tadjikistan or Tadzhik or Tanzania or Thailand or Togo or Togolese Republic or Tonga or Trinidad or Tobago or Tunisia or Turkey or Turkmenistan or Turkmen or Uganda or Ukraine or Uruguay or USSR or Soviet Union or Union of Soviet Socialist Republics or Uzbekistan or Uzbek or Vanuatu or New Hebrides or Venezuela or Vietnam or Viet Nam or West Bank or Yemen or Yugoslavia or Zambia or Zimbabwe or Rhodesia).hw,kf,ti,ab,cp
4. ((developing or less* developed or under developed or underdeveloped or middle income or low* income or underserved or under served or deprived or poor*) adj (countr* or nation? or population? or world)).ti,ab
5. ((developing or less* developed or under developed or underdeveloped or middle income or low* income) adj (economy or economies)).ti,ab
6. (low* adj (gdp or gnp or gross domestic or gross national)).ti,ab
7. (low adj3 middle adj3 countr*).ti,ab.
8. (lmic or lmics or third world or lami countr*).ti,ab.
9. transitional countr*.ti,ab.
10. Or/64-72 (4137)

**RCTs**

1. randomized controlled trial.pt
2. controlled clinical trial.pt.
3. multicenter study.pt.
4. (randomised or randomized or randomly).ti,ab
5. placebo.ti,ab
6. trial.ti,ab
7. groups.ti,ab.
8. intervention.ti,ab.
9. evaluat*.ti,ab
10. control*.ti,ab.
11. effect?.ti,ab.
12. impact.ti,ab
13. (time series or time points).ti,ab.
14. ((pretest or pre test) and (posttest or post test)).ti,ab.
15. (quasi experiment* or quasiexperiment*).ti,ab
16. ((multicentre or multicentre or multi centre or multi center) adj study).ti,ab.
17. Or/ 74-89
18. 5 and 63 and 73 and 90
19. **EMBASE**

**Schizophrenia**

1. exp psychosis/
2. exp schizophrenia/
3. exp bipolar disorder/
4. (Schizophreni* or psychosis or psychoses or psychotic or schizoaffective or Schizophreniform or bipolar disorder or manic depress*).tw.
5. 1 or 2 or 3 or 4

**Psychotherapy**

1. exp psychotherapy/
2. exp counseling/
3. (Psychotherapy or counselling or counseling or therapy or interpersonal therapy or cognitive behavio?ral therapy or art therapy or music therapy or brief intervention or cognitive retraining or cognitive rehabilitation).tw.

**Psychoeducation**

1. health education/ or patient education/ or psychoeducation/
2. (psychoeducation* or psycho-education*).tw.
3. ((Patient* or caregiver* or care-giver* or carer* or family or families) adj3 (education or advice or information or training or support or intervention or livelihood)).tw.

**Family**

1. caregiver burden/ or caregiver/ or caregiver support/
2. family relation/
3. ((family or families or carer or caregiver or care-giver) adj3 burden).tw.

**Adherence**

1. patient compliance/
2. (patient adj3 (compliance or concordance or adherence)).tw
3. ((antipsychotic* or anti-psychotic* or medication*) adj3 (compliance or concordance or adherence or non-compliance or non-concordance or non-adherence)).tw
4. ((Adherence or compliance or medication) adj3 (support or therapy or education or training or advice or information or intervention)).tw.

**Rehabilitation**

1. rehabilitation/ or cognitive rehabilitation/ or community based rehabilitation/ or community reintegration/ or functional training/ or home rehabilitation/ or occupational therapy/ or psychosocial rehabilitation/ or sociotherapy/ or vocational rehabilitation/
2. rehabilitation care/ or rehabilitation center/
3. rehabilitation nursing/
4. social adaptation/
5. human relation/ or social network/
6. social isolation/
7. social inclusion.tw.
8. (community based rehabilitation or community-based rehabilitation or CBR).tw.
9. (rehabilitat* adj3 (home based or home-based or communit*)).tw.
10. (Communit* adj3 (vocational training or apprenticeship* or employment placement service* or support network* or self-employ* or supported employ* or social service* or social work*)).tw.
11. (Communit* adj3 (personal assistance or personal assistant* or individual support* or disabled people* organi?ation*)).tw.
12. (Communit* adj3 (empower* or awareness campaign* or self-advocacy or self-help group* or support group* or women* group* or development group*)).tw.
13. (Communit* adj3 inclusi* adj3 (health or education or hous* or social or justice or empower*)).tw.
14. (Rehabilitation adj3 (vocational or social or personal)).tw
15. (Training adj3 (Life skill* or social skill* or personal skill* or interpersonal skill* or interpersonal)).tw.
16. ((Psychosocial or psycho-social or social or psychological or psychiatric or PSR or vocational or occupational) adj3 (intervention* or support or rehabilitation)).tw.
17. ((Sustainable livelihood* or livelihood*) adj3 (intervention or support)).tw.
18. (Recovery or recovery model or recovery approach or social recovery).tw.

**Health promotion**

1. health promotion/
2. (Health adj3 (promotion or advice or information or training or support)).tw.

**Self help**

1. social support/
2. exp self help/
3. ((Social or peer* or peer-led or peer led or self help or self-help or community or cooperative or co-operative) adj3 (group or support or support group)).tw.

**Collaborative/ community/ primary care**

1. (care adj3 (Collaborative or community or community-based)).tw.
2. (outreach adj3 (service* or care or intervention* or program*)).tw.
3. community care/ or community health nursing/ or community program/
4. home care/ or home mental health care/
5. mental health service/
6. social work/
7. primary health care/
8. health auxiliary/
9. outpatient care/
10. ambulatory care/ or ambulatory care nursing/
11. paramedical personnel/
12. ((nonspecialist* or non-specialist* or allied health or nurse led or community health or village health) adj3 (worker* or personnel or team*)).tw.
13. or/6-53

**Low and middle income countries**

1. developing country/
2. (Africa or Asia or Caribbean or West Indies or South America or Latin America or Central America).cp,ti,ab,sh,hw,kw
3. (Afghanistan or Albania or Algeria or Angola or Antigua or Barbuda or Argentina or Armenia or Armenian or Aruba or Azerbaijan or Bahrain or Bangladesh or Barbados or Benin or Byelarus or Byelorussian or Belarus or Belorussian or Belorussia or Belize or Bhutan or Bolivia or Bosnia or Herzegovina or Hercegovina or Botswana or Brazil or Bulgaria or Burkina Faso or Burkina Fasso or Upper Volta or Burundi or Urundi or Cambodia or Khmer Republic or Kampuchea or Cameroon or Cameroons or Cameron or Camerons or Cape Verde or Central African Republic or Chad or Chile or China or Colombia or Comoros or Comoro Islands or Comores or Mayotte or Congo or Zaire or Costa Rica or Cote Ivoire or Ivory Coast or Croatia or Cuba or Cyprus or Czechoslovakia or Czech Republic or Slovakia or Slovak Republic or Djibouti or French Somaliland or Dominica or Dominican Republic or East Timor or East Timur or Timor Leste or Ecuador or Egypt or United Arab Republic or El Salvador or Eritrea or Estonia or Ethiopia or Fiji or Gabon or Gabonese Republic or Gambia or Gaza or Georgia Republic or Georgian Republic or Ghana or Gold Coast or Greece or Grenada or Guatemala or Guinea or Guam or Guiana or Guyana or Haiti or Honduras or Hungary or India or Maldives or Indonesia or Iran or Iraq or Isle of Man or Jamaica or Jordan or Kazakhstan or Kazakh or Kenya or Kiribati or Korea or Kosovo or Kyrgyzstan or Kirghizia or Kyrgyz Republic or Kirghiz or Kirgizstan or Lao PDR or Laos or Latvia or Lebanon or Lesotho or Basutoland or Liberia or Libya or Lithuania or Macedonia or Madagascar or Malagasy Republic or Malaysia or Malaya or Malay or Sabah or Sarawak or Malawi or Nyasaland or Mali or Malta or Marshall Islands or Mauritania or Mauritius or Agalega Islands or Mexico or Micronesia or Middle East or Moldova or Moldovia or Moldovian or Mongolia or Montenegro or Morocco or Ifni or Mozambique or Myanmar or Myanma or Burma or Namibia or Nepal or Netherlands Antilles or New Caledonia or Nicaragua or Niger or Nigeria or Northern Mariana Islands or Oman or Muscat or Pakistan or Palau or Palestine or Panama or Paraguay or Peru or Philippines or Philipines or Phillipines or Phillippines or Poland or Portugal or Puerto Rico or Romania or Rumania or Roumania or Russia or Russian or Rwanda or Ruanda or Saint Kitts or St Kitts or Nevis or Saint Lucia or St Lucia or Saint Vincent or St Vincent or Grenadines or Samoa or Samoan Islands or Navigator Island or Navigator Islands or Sao Tome or Saudi Arabia or Senegal or Serbia or Montenegro or Seychelles or Sierra Leone or Slovenia or Sri Lanka or Ceylon or Solomon Islands or Somalia or Sudan or Suriname or Surinam or Swaziland or Syria or Tajikistan or Tadzhikistan or Tadjikistan or Tadzhik or Tanzania or Thailand or Togo or Togolese Republic or Tonga or Trinidad or Tobago or Tunisia or Turkey or Turkmenistan or Turkmen or Uganda or Ukraine or Uruguay or USSR or Soviet Union or Union of Soviet Socialist Republics or Uzbekistan or Uzbek or Vanuatu or New Hebrides or Venezuela or Vietnam or Viet Nam or West Bank or Yemen or Yugoslavia or Zambia or Zimbabwe or Rhodesia).cp,ti,sh,hw,ab,kw.
4. ((developing or less* developed or under developed or underdeveloped or middle income or low* income or underserved or under served or deprived or poor*) adj (countr* or nation? or population? or world)).ti,ab.
5. ((developing or less* developed or under developed or underdeveloped or middle income or low* income) adj (economy or economies)).ti,ab.
6. (low* adj (gdp or gnp or gross domestic or gross national)).ti,ab.
7. (low adj3 middle adj3 countr*).ti,ab.
8. (lmic or lmics or third world or lami countr*).ti,ab.
9. transitional countr*.ti,ab.
10. or/55-63
11. 5 and 54 and 64 (5518)
12. **PSYCHinfo**

**Schizophrenia**

1. psychosis/ or affective psychosis/ or chronic psychosis/ or "paranoia (psychosis)"/ or postpartum psychosis/ or exp schizophrenia/ or paranoid schizophrenia/
2. exp bipolar disorder/
3. (Schizophreni* or psychosis or psychoses or psychotic or schizoaffective or Schizophreniform or bipolar disorder or manic depress*).tw.
4. 1 or 2 or 3

**Psychotherapy**

1. exp psychotherapy/
2. exp counseling/
3. (Psychotherapy or counselling or counseling or therapy or interpersonal therapy or cognitive behavio?ral therapy or art therapy or music therapy or brief intervention or cognitive retraining or cognitive rehabilitation).tw.

**Psychoeducation**

1. health education/ or psychoeducation/
2. client education/
3. (psychoeducation* or psycho-education*).tw.
4. ((Patient* or caregiver* or care-giver* or carer* or family or families) adj3 (education or advice or information or training or support or intervention or livelihood)).tw.

**Family**

1. exp family therapy/ or family intervention/
2. caregivers/ or caregiver burden/
3. ((family or families or carer or caregiver or care-giver) adj3 burden).tw.

**Adherence**

1. treatment compliance/ or treatment barriers/ or treatment dropouts/
2. (patient adj3 (compliance or concordance or adherence)).tw.
3. ((antipsychotic* or anti-psychotic* or medication*) adj3 (compliance or concordance or adherence or non-compliance or non-concordance or non-adherence)).tw.
4. ((Adherence or compliance or medication) adj3 (support or therapy or education or training or advice or information or intervention)).tw.

**Rehabilitation**

1. exp rehabilitation/ or exp psychosocial rehabilitation/
2. disability management/
3. exp rehabilitation centers/
4. social adjustment/
5. exp interpersonal relationships/ or interpersonal interaction/
6. social isolation/
7. social integration/
8. (community based rehabilitation or community-based rehabilitation or CBR).tw.
9. (rehabilitat* adj3 (home based or home-based or communit*)).tw.
10. (Communit* adj3 (vocational training or apprenticeship* or employment placement service* or support network* or self-employ* or supported employ* or social service* or social work*)).tw.
11. (Communit* adj3 (personal assistance or personal assistant* or individual support* or disabled people* organi?ation*)).tw.
12. (Communit* adj3 (empower* or awareness campaign* or self-advocacy or self-help group* or support group* or women* group* or development group*)).tw.
13. (Communit* adj3 inclusi* adj3 (health or education or hous* or social or justice or empower*)).tw.
14. (Rehabilitation adj3 (vocational or social or personal)).tw.
15. (Training adj3 (Life skill* or social skill* or personal skill* or interpersonal skill* or interpersonal)).tw.
16. ((Psychosocial or psycho-social or social or psychological or psychiatric or PSR or vocational or occupational) adj3 (intervention* or support or rehabilitation)).tw.
17. ((Sustainable livelihood* or livelihood*) adj3 (intervention or support)).tw.
18. (Recovery or recovery model or recovery approach or social recovery).tw.
19. "recovery (disorders)"/

**Health promotion**

1. health promotion/
2. (Health adj3 (promotion or advice or information or training or support)).tw.

**Self help**

1. social support/ or social networks/ or support groups/
2. exp self help techniques/
3. ((Social or peer* or peer-led or peer led or self help or self-help or community or cooperative or co-operative) adj3 (group or support or support group)).tw.

**Collaborative/ community/ primary care**

1. (care adj3 (Collaborative or community or community-based)).tw.
2. (outreach adj3 (service* or care or intervention* or program*)).tw.
3. deinstitutionalization/ or community mental health/ or exp community mental health services/
4. home care/
5. community services/ or home visiting programs/
6. outreach programs/
7. primary health care/
8. interdisciplinary treatment approach/
9. exp social casework/
10. exp allied health personnel/
11. outpatients/ or outpatient treatment/
12. ((nonspecialist* or non-specialist* or allied health or nurse led or community health or village health) adj3 (worker* or personnel or team*)).tw.

**Low and middle income countries**

1. exp Developing Countries/
2. (Africa or Asia or Caribbean or West Indies or South America or Latin America or Central America).ab,hw,lo,pl,sh,ti.
3. (Afghanistan or Albania or Algeria or Angola or Antigua or Barbuda or Argentina or Armenia or Armenian or Aruba or Azerbaijan or Bahrain or Bangladesh or Barbados or Benin or Byelarus or Byelorussian or Belarus or Belorussian or Belorussia or Belize or Bhutan or Bolivia or Bosnia or Herzegovina or Hercegovina or Botswana or Brazil or Bulgaria or Burkina Faso or Burkina Fasso or Upper Volta or Burundi or Urundi or Cambodia or Khmer Republic or Kampuchea or Cameroon or Cameroons or Cameron or Camerons or Cape Verde or Central African Republic or Chad or Chile or China or Colombia or Comoros or Comoro Islands or Comores or Mayotte or Congo or Zaire or Costa Rica or Cote Ivoire or Ivory Coast or Croatia or Cuba or Cyprus or Czechoslovakia or Czech Republic or Slovakia or Slovak Republic or Djibouti or French Somaliland or Dominica or Dominican Republic or East Timor or East Timur or Timor Leste or Ecuador or Egypt or United Arab Republic or El Salvador or Eritrea or Estonia or Ethiopia or Fiji or Gabon or Gabonese Republic or Gambia or Gaza or Georgia Republic or Georgian Republic or Ghana or Gold Coast or Greece or Grenada or Guatemala or Guinea or Guam or Guiana or Guyana or Haiti or Honduras or Hungary or India or Maldives or Indonesia or Iran or Iraq or Isle of Man or Jamaica or Jordan or Kazakhstan or Kazakh or Kenya or Kiribati or Korea or Kosovo or Kyrgyzstan or Kirghizia or Kyrgyz Republic or Kirghiz or Kirgizstan or Lao PDR or Laos or Latvia or Lebanon or Lesotho or Basutoland or Liberia or Libya or Lithuania or Macedonia or Madagascar or Malagasy Republic or Malaysia or Malaya or Malay or Sabah or Sarawak or Malawi or Nyasaland or Mali or Malta or Marshall Islands or Mauritania or Mauritius or Agalega Islands or Mexico or Micronesia or Middle East or Moldova or Moldovia or Moldovian or Mongolia or Montenegro or Morocco or Ifni or Mozambique or Myanmar or Myanma or Burma or Namibia or Nepal or Netherlands Antilles or New Caledonia or Nicaragua or Niger or Nigeria or Northern Mariana Islands or Oman or Muscat or Pakistan or Palau or Palestine or Panama or Paraguay or Peru or Philippines or Philipines or Phillipines or Phillippines or Poland or Portugal or Puerto Rico or Romania or Rumania or Roumania or Russia or Russian or Rwanda or Ruanda or Saint Kitts or St Kitts or Nevis or Saint Lucia or St Lucia or Saint Vincent or St Vincent or Grenadines or Samoa or Samoan Islands or Navigator Island or Navigator Islands or Sao Tome or Saudi Arabia or Senegal or Serbia or Montenegro or Seychelles or Sierra Leone or Slovenia or Sri Lanka or Ceylon or Solomon Islands or Somalia or Sudan or Suriname or Surinam or Swaziland or Syria or Tajikistan or Tadzhikistan or Tadjikistan or Tadzhik or Tanzania or Thailand or Togo or Togolese Republic or Tonga or Trinidad or Tobago or Tunisia or Turkey or Turkmenistan or Turkmen or Uganda or Ukraine or Uruguay or USSR or Soviet Union or Union of Soviet Socialist Republics or Uzbekistan or Uzbek or Vanuatu or New Hebrides or Venezuela or Vietnam or Viet Nam or West Bank or Yemen or Yugoslavia or Zambia or Zimbabwe or Rhodesia).ab,hw,lo,ti,sh,pl.
4. ((developing or less* developed or under developed or underdeveloped or middle income or low* income or underserved or under served or deprived or poor*) adj (countr* or nation? or population? or world)).tw.
5. ((developing or less* developed or under developed or underdeveloped or middle income or low* income) adj (economy or economies)).ti,ab.
6. (low* adj (gdp or gnp or gross domestic or gross national)).ti,ab.
7. (low adj3 middle adj3 countr*).ti,ab.
8. (lmic or lmics or third world or lami countr*).ti,ab.
9. transitional countr*.ti,ab.
10. or/55-63
11. **GLOBAL HEALTH**

**Schizophrenia**

1. psychoses/ or schizophrenia/
2. bipolar disorder/
3. (Schizophreni* or psychosis or psychoses or psychotic or schizoaffective or Schizophreniform or bipolar disorder or manic depress*).tw
4. 1 or 2 or 3

**Psychotherapy**

1. psychotherapy/
2. exp counselling/
3. (Psychotherapy or counselling or counseling or therapy or interpersonal therapy or cognitive behavio?ral therapy or art therapy or music therapy or brief intervention or cognitive retraining or cognitive rehabilitation).tw.

**Psychoeducation**

1. health education/
2. patient education/
3. (psychoeducation* or psycho-education*).tw.

**Family**

1. ((Patient* or caregiver* or care-giver* or carer* or family or families) adj3 (education or advice or information or training or support or intervention or livelihood)).tw.
2. ((family or families or carer or caregiver or care-giver) adj3 burden).tw.

**Adherence**

1. patient compliance/
2. (patient adj3 (compliance or concordance or adherence)).tw.
3. ((antipsychotic* or anti-psychotic* or medication*) adj3 (compliance or concordance or adherence or non-compliance or non-concordance or non-adherence)).tw.
4. ((Adherence or compliance or medication) adj3 (support or therapy or education or training or advice or information or intervention)).tw.

**Rehabilitation**

1. rehabilitation/
2. social adjustment/ or social integration/
3. interpersonal relations/ or social participation/
4. social inclusion.tw.
5. (community based rehabilitation or community-based rehabilitation or CBR).tw.
6. (rehabilitat* adj3 (home based or home-based or communit*)).tw.
7. (Communit* adj3 (vocational training or apprenticeship* or employment placement service* or support network* or self-employ* or supported employ* or social service* or social work*)).tw.
8. (Communit* adj3 (personal assistance or personal assistant* or individual support* or disabled people* organi?ation*)).tw.
9. (Communit* adj3 (empower* or awareness campaign* or self-advocacy or self-help group* or support group* or women* group* or development group*)).tw.
10. (Communit* adj3 inclusi* adj3 (health or education or hous* or social or justice or empower*)).tw.
11. (Rehabilitation adj3 (vocational or social or personal)).tw.
12. (Training adj3 (Life skill* or social skill* or personal skill* or interpersonal skill* or interpersonal)).tw.
13. ((Psychosocial or psycho-social or social or psychological or psychiatric or PSR or vocational or occupational) adj3 (intervention* or support or rehabilitation)).tw.
14. ((Sustainable livelihood* or livelihood*) adj3 (intervention or support)).tw.
15. (Recovery or recovery model or recovery approach or social recovery).tw.

**Health promotion**

1. health promotion/
2. (Health adj3 (promotion or advice or information or training or support)).tw.

**Self help**

1. self help/ or community development/ or self care/
2. ((Social or peer* or peer-led or peer led or self help or self-help or community or cooperative or co-operative) adj3 (group or support or support group)).tw.

**Collaborative/ community/ primary care**

1. (care adj3 (Collaborative or community or community-based)).tw.
2. (outreach adj3 (service* or care or intervention* or program*)).tw.
3. community health services/ or community health/ or community programmes/
4. community health services/ or community health/ or community involvement/ or community programmes/
5. home care/
6. social services/ or social workers/
7. outpatient services/
8. primary health care/
9. medical auxiliaries/
10. ((nonspecialist* or non-specialist* or allied health or nurse led or community health or village health) adj3 (worker* or personnel or team*)).tw.
11. or/5-45

**Developing countries**

1. Developing Countries/
2. (Africa or Asia or Caribbean or West Indies or South America or Latin America or Central America).ab,cp,gl,hw,sh,ti.
3. (Afghanistan or Albania or Algeria or Angola or Antigua or Barbuda or Argentina or Armenia or Armenian or Aruba or Azerbaijan or Bahrain or Bangladesh or Barbados or Benin or Byelarus or Byelorussian or Belarus or Belorussian or Belorussia or Belize or Bhutan or Bolivia or Bosnia or Herzegovina or Hercegovina or Botswana or Brazil or Bulgaria or Burkina Faso or Burkina Fasso or Upper Volta or Burundi or Urundi or Cambodia or Khmer Republic or Kampuchea or Cameroon or Cameroons or Cameron or Camerons or Cape Verde or Central African Republic or Chad or Chile or China or Colombia or Comoros or Comoro Islands or Comores or Mayotte or Congo or Zaire or Costa Rica or Cote Ivoire or Ivory Coast or Croatia or Cuba or Cyprus or Czechoslovakia or Czech Republic or Slovakia or Slovak Republic or Djibouti or French Somaliland or Dominica or Dominican Republic or East Timor or East Timur or Timor Leste or Ecuador or Egypt or United Arab Republic or El Salvador or Eritrea or Estonia or Ethiopia or Fiji or Gabon or Gabonese Republic or Gambia or Gaza or Georgia Republic or Georgian Republic or Ghana or Gold Coast or Greece or Grenada or Guatemala or Guinea or Guam or Guiana or Guyana or Haiti or Honduras or Hungary or India or Maldives or Indonesia or Iran or Iraq or Isle of Man or Jamaica or Jordan or Kazakhstan or Kazakh or Kenya or Kiribati or Korea or Kosovo or Kyrgyzstan or Kirghizia or Kyrgyz Republic or Kirghiz or Kirgizstan or Lao PDR or Laos or Latvia or Lebanon or Lesotho or Basutoland or Liberia or Libya or Lithuania or Macedonia or Madagascar or Malagasy Republic or Malaysia or Malaya or Malay or Sabah or Sarawak or Malawi or Nyasaland or Mali or Malta or Marshall Islands or Mauritania or Mauritius or Agalega Islands or Mexico or Micronesia or Middle East or Moldova or Moldovia or Moldovian or Mongolia or Montenegro or Morocco or Ifni or Mozambique or Myanmar or Myanma or Burma or Namibia or Nepal or Netherlands Antilles or New Caledonia or Nicaragua or Niger or Nigeria or Northern Mariana Islands or Oman or Muscat or Pakistan or Palau or Palestine or Panama or Paraguay or Peru or Philippines or Philipines or Phillipines or Phillippines or Poland or Portugal or Puerto Rico or Romania or Rumania or Roumania or Russia or Russian or Rwanda or Ruanda or Saint Kitts or St Kitts or Nevis or Saint Lucia or St Lucia or Saint Vincent or St Vincent or Grenadines or Samoa or Samoan Islands or Navigator Island or Navigator Islands or Sao Tome or Saudi Arabia or Senegal or Serbia or Montenegro or Seychelles or Sierra Leone or Slovenia or Sri Lanka or Ceylon or Solomon Islands or Somalia or Sudan or Suriname or Surinam or Swaziland or Syria or Tajikistan or Tadzhikistan or Tadjikistan or Tadzhik or Tanzania or Thailand or Togo or Togolese Republic or Tonga or Trinidad or Tobago or Tunisia or Turkey or Turkmenistan or Turkmen or Uganda or Ukraine or Uruguay or USSR or Soviet Union or Union of Soviet Socialist Republics or Uzbekistan or Uzbek or Vanuatu or New Hebrides or Venezuela or Vietnam or Viet Nam or West Bank or Yemen or Yugoslavia or Zambia or Zimbabwe or Rhodesia).ab,cp,gl,hw,sh,ti.
4. ((developing or less* developed or under developed or underdeveloped or middle income or low* income or underserved or under served or deprived or poor*) adj (countr* or nation? or population? or world)).ti,ab.
5. ((developing or less* developed or under developed or underdeveloped or middle income or low* income) adj (economy or economies)).ti,ab.
6. (low* adj (gdp or gnp or gross domestic or gross national)).ti,ab.
7. (low adj3 middle adj3 countr*).ti,ab.
8. (lmic or lmics or third world or lami countr*).ti,ab.
9. transitional countr*.ti,ab.
10. or/47-55
11. 4 and 46 and 56
12. **CINAHL (952)**

**Schizophrenia**

1. (MH "Schizophrenia+") OR (MH "Psychotic Disorders") OR (MH "Schizoaffective Disorder")
2. (MH "Bipolar Disorder+") OR (MH "Affective Disorders, Psychotic+")
3. Schizophreni* or psychosis or psychoses or psychotic or schizoaffective or Schizophreniform or bipolar disorder or manic depress* or psychotic affective disorder
4. S1 OR S2 OR S3

**Psychotherapy**

1. (MH "Psychotherapy+") OR (MH “Psychosocial support”)
2. Psychotherapy or counselling or counseling or therapy or interpersonal therapy or cognitive behavio?ral therapy or art therapy or music therapy or brief intervention or cognitive retraining or cognitive rehabilitation
3. (MH "Health education") OR (MH “patient education”)
4. Psychoeducation* or psycho-education* or Patient* information or patient advice or patient support or livelihood support or carer* advice or caregiver* advice or carer information or caregiver* information or carer* training or caregiver* training or carer* support or caregiver* support or carer* intervention* or caregiver* intervention* or famil* education or famil* information or famil* support or famil* intervention or famil* burden or Caregiver* burden or carer* burden

**Family**

1. (MH "Caregiver Burden") OR (MH "Caregivers") OR (MH "Caregiver Support")
2. (MH "Family") OR (MH "Family Coping")

**Adherence**

1. (MH "Patient Compliance+")
2. Adherence or concordance or non-compliance or non-concordance or non-adherence or adherence support or adherence therapy or adherence training or adherence advice or adherence intervention or compliance support or compliance therapy or compliance training or compliance advice or compliance intervention

**Rehabilitation**

1. (MH "Rehabilitation") OR (MH "Activities of Daily Living+") OR (MH "Home Rehabilitation") OR (MH "Occupational Therapy") OR (MH "Rehabilitation, Psychosocial+") OR (MH "Rehabilitation, Vocational") OR (MH "Rehabilitation, Community-Based") OR (MH "Rehabilitation, Cognitive") (MH "Home Health Care") OR (MH "Psychiatric Home Care") OR (MH "Home Rehabilitation")
2. (MH "Interpersonal Relations") OR (MH "Patient-Family Relations") OR (MH "Psychosocial Aspects of Illness") OR (MH "Social Isolation") OR (MH "Social Behavior") OR (MH "Social Adjustment") OR (MH "Social Skills") OR (MH "Social Participation") OR (MH "Symptom Distress") OR (MH "Psychosocial Deprivation")
3. CBR or community based rehabilitation or community-based rehabiltiation or community empowerment or self advocacy or occupational rehabilitation or vocational rehabilitation or personal rehabilitation or social rehabilitation or psychosocial rehabilitation or psychiatric rehabilitation or PSR or home based rehabilitation or vocational training or community support
4. Social inclusion or social skill* training or life skill* training or interpersonal training or psychosocial intervention or livelihood intervention or livelihood support or recovery or support network or social service or social work

**Health Promotion**

1. (MH "Health Promotion")
2. Health promotion or Health advice or Health information

**Self help**

1. (MH "Peer Group") OR (MH "Support Groups")
2. peer* support or peer support group or self-help group or self help group or community support group or cooperative group or co-operative group or community group

**Collaborative/ community/ primary care**

1. (MH "Community Mental Health Services+") OR (MH "Community Health Services") OR (MH "Community Health Centers") OR (MH "Community Networks") OR (MH "Home Health Care")
2. (MH "Social Work+")
3. (MH "Primary Health Care") OR (MH "Multidisciplinary Care Team") OR (MH "Community Health Nursing+") OR (MH "Rehabilitation Nursing") OR (MH "Psychiatric Nursing") OR (MH "Community Mental Health Nursing")
4. (MH "Rehabilitation Patients") OR (MH "Outpatients")
5. (MH "Ambulatory Care")
6. (MH "Outpatient Service") OR (MH "Social Work Service")
7. (MH "Community Health Workers") OR (MH "Health Educators") OR (MH "Allied Health Personnel") OR (MH "Social Workers") OR (MH "Rural Health Personnel")
8. nonspecialist* or non-specialist* or allied health worker* or nurse led or community health worker* or village health worker* or lay health worker* or collaborative care or community care or community-based care or outreach service* or outreach program* or outreach intervention*
9. S5 OR S6 OR S7 OR S8 OR S9 OR S10 OR S11 OR S12 OR S13 OR S14 OR S15 OR S16 OR S17 OR S18 OR S19 OR S20 OR S21 OR S22 OR S23 OR S24 OR S25 OR S26 OR S27 OR S28

**Low and middle income countries**

1. (MH "Developing Countries") OR (MH "Africa") OR (MH "Central America+") OR (MH "Latin America") OR (MH "South America") OR (MH "Asia+") OR (MH "Europe, Eastern") OR (MH "Indian Ocean Islands+") OR (MH "Pacific Islands") OR (MH "Melanesia+") OR (MH "Micronesia+") OR (MH "Polynesia+")
2. Africa or Asia or Caribbean or West Indies or South America or Latin America or Central America
3. Afghanistan or Albania or Algeria or Angola or Antigua or Barbuda or Argentina or Armenia or Armenian or Aruba or Azerbaijan or Bahrain or Bangladesh or Barbados or Benin or Byelarus or Byelorussian or Belarus or Belorussian or Belorussia or Belize or Bhutan or Bolivia or Bosnia or Herzegovina or Hercegovina or Botswana or Brazil or Bulgaria or Burkina Faso or Burkina Fasso or Upper Volta or Burundi or Urundi or Cambodia or Khmer Republic or Kampuchea or Cameroon or Cameroons or Cameron or Camerons or Cape Verde or Central African Republic or Chad or Chile or China or Colombia or Comoros or Comoro Islands or Comores or Mayotte or Congo or Zaire or Costa Rica or Cote Ivoire or Ivory Coast or Croatia or Cuba or Cyprus or Czechoslovakia or Czech Republic or Slovakia or Slovak Republic or Djibouti or French Somaliland or Dominica or Dominican Republic or East Timor or East Timur or Timor Leste or Ecuador or Egypt or United Arab Republic or El Salvador or Eritrea or Estonia or Ethiopia or Fiji or Gabon or Gabonese Republic or Gambia or Gaza or Georgia Republic or Georgian Republic or Ghana or Gold Coast or Greece or Grenada or Guatemala or Guinea or Guam or Guiana or Guyana or Haiti or Honduras or Hungary or India or Maldives or Indonesia or Iran or Iraq or Isle of Man or Jamaica or Jordan or Kazakhstan or Kazakh or Kenya or Kiribati or Korea or Kosovo or Kyrgyzstan or Kirghizia or Kyrgyz Republic or Kirghiz or Kirgizstan or Lao PDR or Laos or Latvia or Lebanon or Lesotho or Basutoland or Liberia or Libya or Lithuania or Macedonia or Madagascar or Malagasy Republic or Malaysia or Malaya or Malay or Sabah or Sarawak or Malawi or Nyasaland or Mali or Malta or Marshall Islands or Mauritania or Mauritius or Agalega Islands or Mexico or Micronesia or Middle East or Moldova or Moldovia or Moldovian or Mongolia or Montenegro or Morocco or Ifni or Mozambique or Myanmar or Myanma or Burma or Namibia or Nepal or Netherlands Antilles or New Caledonia or Nicaragua or Niger or Nigeria or Northern Mariana Islands or Oman or Muscat or Pakistan or Palau or Palestine or Panama or Paraguay or Peru or Philippines or Philipines or Phillipines or Phillippines or Poland or Portugal or Puerto Rico or Romania or Rumania or Roumania or Russia or Russian or Rwanda or Ruanda or Saint Kitts or St Kitts or Nevis or Saint Lucia or St Lucia or Saint Vincent or St Vincent or Grenadines or Samoa or Samoan Islands or Navigator Island or Navigator Islands or Sao Tome or Saudi Arabia or Senegal or Serbia or Montenegro or Seychelles or Sierra Leone or Slovenia or Sri Lanka or Ceylon or Solomon Islands or Somalia or Sudan or Suriname or Surinam or Swaziland or Syria or Tajikistan or Tadzhikistan or Tadjikistan or Tadzhik or Tanzania or Thailand or Togo or Togolese Republic or Tonga or Trinidad or Tobago or Tunisia or Turkey or Turkmenistan or Turkmen or Uganda or Ukraine or Uruguay or USSR or Soviet Union or Union of Soviet Socialist Republics or Uzbekistan or Uzbek or Vanuatu or New Hebrides or Venezuela or Vietnam or Viet Nam or West Bank or Yemen or Yugoslavia or Zambia or Zimbabwe or Rhodesia
4. developing country or developing world or low income countr* or middle income countr* or poor countr* or deprived countr* or LMIC* or third world
5. S30 OR S31 OR S32 OR S33
6. S4 AND S29 AND S34
7. **THE COCHRANE LIBRARY**

**(130 trials including steps 1-3, 110 cochrane reviews including steps 1-2 and 46 other reviews including steps 1-2)**

**Nb Developing countries terms (step 3) excluded from search of reviews as Cochrane reviews do not limit by country and may include a mixture of papers from HIC and LMIC**

1. schizophrenia OR "bipolar disorder" OR psychosis

AND

1. “psychosocial” OR “psychotherapy” OR “counsel?ing” OR “interpersonal therapy” OR “cognitive behavio?ral therapy” OR “art therapy” OR “music therapy” OR “brief intervention” OR “cognitive retraining” OR “cognitive rehabilitation” OR “cognitive remediation” OR “health education” OR “patient education” OR “patient advice” OR “patient information” OR “carer information” OR “carergiver information” OR “carer support” OR “caregiver support” OR “famil* support” OR “famil* intervention” OR “adherence support” OR “adherence therapy” OR “adherence intervention” OR “compliance support” OR “compliance therapy” OR “compliance intervention” OR “rehabilitation” OR “community based rehabilitation” OR “community-based rehabilitation” OR “vocational rehabilitation” OR “social rehabilitation” OR “personal rehabilitation” OR “Psychosocial rehabilitation” OR “psycho-social rehabilitation” OR “social rehabilitation” OR “psychological rehabilitation” OR “psychiatric rehabilitation” OR “PSR” OR “vocational rehabilitation” OR “occupational rehabilitation” OR “psychosocial intervention” OR “psychosocial support” OR “recovery” OR “Life skill* training” OR “social skill* training” OR “personal skill* training” OR “interpersonal skill* training” OR “interpersonal training” OR “health promotion” OR “health advice” OR “health information” OR “social support” OR “self help group” OR “self-help group” OR “peer led group” OR “peer support” OR “peer-led support” OR “community support” OR “collaborative care” OR “community care” OR “community-based care” OR “outreach service” OR “outreach intervention” OR “outreach program” OR “community health service” OR “home nursing” OR “home care” OR “primary care” OR “community mental health service” OR “social work*” OR “community health worker” OR “village health worker” OR “lay health worker” OR “non-specialist” OR “nonspecialist” OR “auxillar*” OR “allied health personnel” OR “nurse led” OR “nurse-led”

AND

1. “developing countr*” or Africa or Asia or Caribbean or “West Indies” or “South America” or “Latin America” or “Central America” or Afghanistan or Albania or Algeria or Angola or Antigua or Barbuda or Argentina or Armenia or Armenian or Aruba or Azerbaijan or Bahrain or Bangladesh or Barbados or Benin or Byelarus or Byelorussian or Belarus or Belorussian or Belorussia or Belize or Bhutan or Bolivia or Bosnia or Herzegovina or Hercegovina or Botswana or Brazil or Bulgaria or “Burkina Faso” or “Burkina Fasso” or Upper Volta or Burundi or Urundi or Cambodia or Khmer Republic or Kampuchea or Cameroon or Cameroons or Cameron or Camerons or Cape Verde or Central African Republic or Chad or Chile or China or Colombia or Comoros or Comoro Islands or Comores or Mayotte or Congo or Zaire or Costa Rica or Cote Ivoire or Ivory Coast or Croatia or Cuba or Cyprus or Czechoslovakia or Czech Republic or Slovakia or Slovak Republic or Djibouti or French Somaliland or Dominica or Dominican Republic or East Timor or “East Timur” or “Timor Leste” or Ecuador or Egypt or “United Arab Republic” or “El Salvador” or Eritrea or Estonia or Ethiopia or Fiji or Gabon or “Gabonese Republic” or Gambia or Gaza or “Georgia Republic” or “Georgian Republic” or Ghana or “Gold Coast” or Greece or Grenada or Guatemala or Guinea or Guam or Guiana or Guyana or Haiti or Honduras or Hungary or India or Maldives or Indonesia or Iran or Iraq or Isle of Man or Jamaica or Jordan or Kazakhstan or Kazakh or Kenya or Kiribati or Korea or Kosovo or Kyrgyzstan or Kirghizia or Kyrgyz Republic or Kirghiz or Kirgizstan or Lao PDR or Laos or Latvia or Lebanon or Lesotho or Basutoland or Liberia or Libya or Lithuania or Macedonia or Madagascar or Malagasy Republic or Malaysia or Malaya or Malay or Sabah or Sarawak or Malawi or Nyasaland or Mali or Malta or Marshall Islands or Mauritania or Mauritius or Agalega Islands or Mexico or Micronesia or Middle East or Moldova or Moldovia or Moldovian or Mongolia or Montenegro or Morocco or Ifni or Mozambique or Myanmar or Myanma or Burma or Namibia or Nepal or Netherlands Antilles or New Caledonia or Nicaragua or Niger or Nigeria or Northern Mariana Islands or Oman or Muscat or Pakistan or Palau or Palestine or Panama or Paraguay or Peru or Philippines or Philipines or Phillipines or Phillippines or Poland or Portugal or Puerto Rico or Romania or Rumania or Roumania or Russia or Russian or Rwanda or Ruanda or Saint Kitts or St Kitts or Nevis or Saint Lucia or St Lucia or Saint Vincent or St Vincent or Grenadines or Samoa or Samoan Islands or Navigator Island or Navigator Islands or Sao Tome or Saudi Arabia or Senegal or Serbia or Montenegro or Seychelles or Sierra Leone or Slovenia or Sri Lanka or Ceylon or Solomon Islands or Somalia or Sudan or Suriname or Surinam or Swaziland or Syria or Tajikistan or Tadzhikistan or Tadjikistan or Tadzhik or Tanzania or Thailand or Togo or Togolese Republic or Tonga or Trinidad or Tobago or Tunisia or Turkey or Turkmenistan or Turkmen or Uganda or Ukraine or Uruguay or USSR or Soviet Union or Union of Soviet Socialist Republics or Uzbekistan or Uzbek or Vanuatu or New Hebrides or Venezuela or Vietnam or Viet Nam or West Bank or Yemen or Yugoslavia or Zambia or Zimbabwe or Rhodesia or “less* developed countr*” or “middle income countr*” or “low income countr*” or “deprived countr*” or “poor* countr*” or “third world countr*” or LMIC* or LAMIC*
