# Supplementary material for: Systematic review of feasibility and acceptability of psychosocial interventions for schizophrenia in low and middle income countries
Source: BMC Psychiatry. 2015 Feb 12;15:19. doi: 10.1186/s12888-015-0400-6 (PMC4382830; doi:10.1186/s12888-015-0400-6)
Supplement: Additional file 2: — Data extraction table. [file 12888_2015_400_MOESM2_ESM.doc]

| **STUDY**  **Country (Author, date)** | **Intervention** | **Workforce delivering**  **intervention**  **(specialist/**  **non-specialist)** | **Training & supervision** | **Sample** | **Measures of acceptability and feasibility**  **(e.g. tools, interviews etc)** | **Study design** | **Level of effect (primary outcomes)** | **Method of Analysis for acceptability and feasibility data** | **Quality assess-ment** | **Acceptability**  Any measure or quantitative or qualitative data from service users, care givers or those delivering the intervention showing the acceptability and/or feasibility of the psychosocial interventions. | **Feasibility**  Any measure or quantitative or qualitative data from service users, care givers or those delivering the intervention showing the acceptability and/or feasibility of the psychosocial interventions. |
| --- | --- | --- | --- | --- | --- | --- | --- | --- | --- | --- | --- |
| 1. China  (Xiong, 1994) | 1. Monthly 45 minute counselling sessions with patient and family members in outpatient clinic  2. Family group sessions  Also home visits, individual sessions | Therapists | Not reported | 63 families with member with schizophrenia  34 in intervention group  29 in control group | Compliance with intervention | RCT | 12 month rehospital-isation rates 12.1% in intervention group, 35.7% in control group (p = 0.02)  12 month relapse rate 33.3% in intervention group, 60.7% in control group (p = 0.03) | Proportions reported | Moderate | Compliance with intervention:  Patients  56% - actively compliant (regularly attended appointments)  21% - passively compliant (attended only if reminded)  23%  - non compliant (did not attend and refused home visits)  Family members  41% actively compliant  32% passively compliant  27% non-compliant  Anecdotal description of acceptability – some patients refuse to take treatment or participate in any form of therapy, some family members refuse to accept the illness or drop out when they realise there is no final ‘cure’ |  |
| 2. China  (Ran, 2003) | 1.Monthly family visit – psycho-education  2. Family workshops  3.Crisis intervention | Therapist | Not reported | 357 patients  126 cases in the family intervention  Group  103 cases in the drug group  97 cases in the  control group | Reasons for refusal to participate | RCT | Increased treatment compliance in intervention group (p<0.05).  R elapse rate over 9 months  16.3 % intervention group; 37.8 % drug only group; 61.5 % control group (p<0.05). | Proportions reported | Strong | Of the 8.7% of patients who refused to participate 77.4% did so of because they had no caregiver who could be involved, and 22.6% did so because they were afraid of social stigma |  |
| 3. Poland  (Slupczynka, 1999) | 1.Medication management  2.Individual psychotherapy  3. Daily living and social skills training  4.Therapeutic work with family  5. Welfare assistance | Team – psychiatrist psychologist, 3 nurses, social worker | Training course by Polish and Dutch ‘experts’ | 88 Patients | Treatment satisfaction scale (12 dimensions) | Cohort | Clinically significant improvement in social functioning (20% or greater improvement in functioning) noted in 56% of patients  Rehospital-isations decreased twofold (mean  ±SD = 1.3 ± 1.0 compared with 0.6 -!-- 0.9; paired t = 5.88, df = 87, p < 0.001). | Proportions reported | Moderate | Treatment satisfaction scale (12 dimensions)  Prior to the community team treatment, percentages of patients dissatisfied with particular aspects of care ranged from 56% to 81 %.  Dimensions showing most marked dissatisfaction:  Help in crisis 27.3%  Possibility of receiving help 22.7%  Treatment approach 11.4%  Remaining 9 dimensions on the scale showed less than 10% dissatisfaction |  |
| 4. Turkey  (Tas, 2012) | 1.Family assisted social cognition and interaction training  Family members trained as ‘social cognition partners’  20-week,  manualised group intervention targeting dysfunctional  social cognitive processes | Family member cognition partners | Family members trained by a psychiatrist | 19 patients in intervention group (family members trained)  26 in control (social skills training) group (family members trained) | Likert scale of patient satisfaction with intervention  1=poor  10 = utmost satisfaction | Random-ised pilot study | Large positive effect sizes for intervention group on social functioning  subscales including “social withdrawal” p=0.008, “interpersonal communication” p=0.057,  “prosocial activities” p=0.006,  However, no statistical significance for some other subscales  Total quality of life score better in intervention group (81.17 vs 66.34  p<0.001)  PANSS score 52.51 intervention group, 60.94 in control group (p <0.001) | Satisfaction scale | Strong | Likert scale of patient satisfaction with intervention  1=poor  10 = utmost satisfaction  Mean satisfaction = 8.1 SD = 1.1 |  |
| 5. China  (Zhang, 1993) | 1.Family psycho-education  10 lectures  3 discussions | Lectures delivered by psychologists | Not reported | 3092 patients  Experimental group = 2076 patients  Control group = 1016 patients | Participation rates | Cohort | 12 month Relapse rate 20.4% in intervention group, 31.1% in intervention group (p<0.001)  12 month Rehospitalisation rate 11.7% in intervention group  13% in control group  (p<0.001)  Medication adherence 72.4% in intervention group  69% in control group  (p<0.001) | Proportions reported | Moderate | Rate of participation in each individual session ranged from 77.3 to 99.3%  90.3% of participants attended five or more sessions |  |
| 6. China  (Zhang, 1994) | 1.Family counselling sessions (outpatient clinic)  2.Follow up home visits  3.Family meetings with 15 families every 3 months over 18 months  Topics included; stressful life events, conflict resolution, understanding causes of illness | Counsellors | Not specified | 83 patients and families  Experimental group = 42 patients and family  Control group = 41 patients and family | Description of feasibility issues | Cohort | 18 month Rehospitalisation rate 15.4% in intervention group vs 53.8% in control group (p< 0.01)  Post intervention BPRS 25.5 in intervention group, 30.6 in control group (p<0.05) | n/a | Moderate | The patients acknowledged: (1) the importance of delivered information, (2) an opportunity to  share their experience with the illness with others during the treatment group sessions and (3) better  reconciliation with the fact of being ill. They welcomed even required participation of their relatives in the  programme. | 5 patients dropped out due to moving out of the district or marrying and leaving the parental home  50% of contacts with counsellors for the experimental group were made at home as patients and families did not show up for appointments |
| 7. Egypt, (Gohar, 2013) | 1.Social cognitive training  2 training sessions  per week for 8 weeks | Psychiatrist led groups | In-person, supervised training of the first author from the developers of the programme | 42 patients  22 in intervention group  20 in control group (skills training intervention) | Likert scale of satisfaction with training  1=not at all  10 = very much | RCT | Significant treatment effects on total emotional intelligence scores (F = 24.31, p b .001) and  sub-areas of Identifying Emotions (F = 11.77, p b .001) and Managing Emotions (F = 23.27, p b .001), | Likert scale values | Moderate | Likert scale of satisfaction with training  1=not at all  10 = very much Ratings of  (i)how much they enjoyed the treatment,(ii) how enthusiastic and knowledgeable they found the trainers,  (iii) how effective the training was in helping them deal with daily life.  All scores greater than 8 for intervention and control groups  Attendance levels  (number of session attended out of 16) were also comparable and  relatively high in intervention vs control (M = 13.55; SD = 2.13) and control group (M =  12.90; SD = 2.20) (t = 0.96, p > 0.05). |  |
| 8. Poland (Chadzynska 2011) | 1.Patient group psycho-education sessions | Therapists | Not reported | 167 patients  101 inpatients  50 outpatients (no disaggregation in the analysis) | Questionnaire on subjective opinions on sessions  Questionnaire covered attitudes toward the sessions,  concentration and knowledge about psycho-education.  Assessment of level of difficulty of topics  1– easy  2 – moderately difficult  3- very difficult  Assessment of importance of therapist  qualities  1 - moderate  3 – very important | Cross-sectional | Data on effectiveness of psycho-education not included in study | Proportions, descriptive statistics  Mean scores    Mean scores | Moderate | 84% had positive attitude to the sessions; 12.4% negative  76.9% reported good concentration in sessions;20.7% reported poor concentration  Gaining knowledge about the illness was the most common reason for participation (91 patients, 53.8%).  Knowledge regarded  “course and reasons of illness, pharmacotherapy  rules, learning about oneself, insight and coping with symptoms and generally with illness”.  20 patients (11.8%) assessed that the sessions improve their mood. 11.2% (19 patients) indicated the gains resulting from interactions with other patients during the sessions.  The most difficult topics included: coping with symptoms (2.01), asking for help (2.0), causes of illness (1.94) and noticing the first signs of health state worsening (1.94). Contact with a doctor, pharmacotherapy and avoiding alcohol and narcotics were least difficult, but also least important topics  The most important characteristic was “capable of listening and talking” (2.8)  followed by: trustworthy (2.7), effective (2.7), communicating in a clear and straightforward way (2.7), patient (2.7), having extensive knowledge (2.7)  Patients and therapists were asked about usefulness of different visual aids. Schemes facilitating illness comprehension, photos, brochures, charts with most important information concerning the illness, video materials internet sources, and boards were considered by both patients and therapists to be most helpful. Patients also found scripts including most important information about the illness to be very useful. Task books and tests verifying patients’ knowledge were considered to be relatively least helpful. |  |
| 9. Chile (Cacqueo-Urizar, 2009) | 1.Multifamily intervention  for caregivers of patients with schizophrenia  Weekly sessions  5 modules  18 sessions  (psychoeducation and living skills) | Unclear – centre staffed by psychiatrist, psychologists, social workers, nurses | Not reported | 41 main caregivers (31 females and 10 males)  Intervention group - 18  caregivers  Waitlist group - 23 caregivers | The Family Questionnaire (FQ, Cuevas et al. 1995)  Scores 1-3)  The lower the score, the higher the satisfaction level. | Cross-sectional | Effectiveness data not included in this study of satisfaction with service | Multivariate analysis of variance comparing  satisfaction measure | Strong | The mean level of satisfaction in the total sample was  38.12 (SD = 2.35), indicating that relatives of patients  with schizophrenia feel satisfied with the programme.  The waitlist control group showed a higher level of satisfaction with the Mental Health service than the experimental group (Control: 21.57, SD = .61; Experimental: 22.89, SD = 1.99) A marginally significant difference was observed in satisfaction with the patient’s evolution, with the family intervention presenting higher levels of satisfaction (F = 3.76 P = 0.060; Experimental: 15.22, SD = 2.34; Control: 16.57, SD = 2.08). |  |
| 10. India (Kulhara, 2009) | 1.Manualised psycho-education intervention for carers  10 monthly sessions | Mental health professionals | 2 month training by psychiatrist (lectures and practical training) | 76 patients and caregivers  38 patients and caregivers in both experimental and control groups | Patient Satisfaction Questionnaire, modified for use among caregivers.  Scores ranging from 0 to 12 | RCT | Reduction of  psychopathology (all subscales of the PANSS), ITT analysis  Positive subscale  F = 3.64; df = 8, 592; P < 0.05  Negative subscale F = 5.42; df = 8, 328; P < 0.01  Reduction in disability among patients  Overall disability levels of patient__ F = 12.37; df = 1, 74; P < 0.01 | Descriptive statistics | Strong | Significant increase in carer satisfaction with treatment post intervention  ITT analysis - Satisfaction with treatment 10.2 (SD 2.3) in intervention group; 9 (SD 2.7) in control group t=2.1  Completer analysis - Satisfaction with treatment 11.8 (SD 0.8) in intervention group; 10 (SD 2.6) in control group t=3.3  Caregivers in the structured-intervention group were significantly more likely to be satisfied with the treatment received than caregivers in the routine care group. Caregiver-satisfaction with treatment (t = 2.7; P < 0.01). | Due to involving non-medical personnels, the intervention package was  simple, feasible and not costly. |
| 11. Brazil (Cabral, 2009) | 1.Weekly psycho-education and supportive therapy group for patients  2.Weekly  Psycho-education multi-family group | Not reported | Not reported | 44 caregivers of patients with schizophrenia  40 returned the questionnaire | Evaluation form - adaptation of Anderson *et al*. (1986) ‘Living with schizophrenia evaluation form’  Assess knowledge acquisition and opinion and satisfaction with intervention | Cross-sectional | Approximately two thirds of respondents improved their understanding  of the illness | Percentages reported | Unknown | 85%  found meetings very useful  75% thought they were well organized  82.5% thought enough time for discussion about each subject  99%  believed the meetings helped them to cope with their ill relative  95% approved of the multifamily format. | From discussion: As the group was in the morning, those who worked were unable to attend (although this was a small number as most carers were housewives)  But for each of the  46 patients who complied with the treatment during the study period, at least one relative attended  six or more meetings (total number of meetings not reported). |
| 12. Thailand, (Worakul, 2007) | 1.Family psycho-education programme  1 day programme  Didactic component and group discussion | Psychiatrists | Not reported | 91 caregivers | Evaluation of satisfaction of intervention (instrument not specified, but likely to be custom designed form) | Cohort  Pre/Post quant study | Small statistically significant improvements in knowledge (full score = 10) 6.06 pre intervention vs 6.91 post intervention.  No improvement in Attitude (full score = 60) 37.57 pre-intervention 29.37 post intervention |  | Unknown | Scores – 1 = least satisfied  5= most satisfied  Range of scores 3.76-4.31  Items and scores  (SD)  Interest/Attraction of program 4.17 (0.88)  Usefulness of program 4.10 (0.88)  Suitability of content 3.79 (0.92)  Suitability of media 3.76 (0.85)  Competency of educators 4.31( 0.56)  Suitability of place 4.26 (0.59)  Suitability of timing 3.83 (0.88)  Comprehension 3.93( 0.84)  Suitability of setting 3.83 (0.76) |  |
| 13. Czech Republic (Motlova, 2006) | 1.Outpatient clinic based psycho-education intervention for patients and family  8 hour programme  Parallel sessions for patients and family members | Professionals | Not stated | 93 relatives and 53 patients who participated in the programme were mailed a questionnaire  48.39% relatives and 67.92% of patients returned the questionnaire | Psycho-education Outcomes Questionnaire  (POQ) | Prospective follow up study | Intervention participants had a shorter average length of re-hospitalization  (5.89 vs. 17.78 days, P_/0.045) in a 1-year follow-up after discharge. | Detailed quantitative and qualitative analysis is reported in another paper (not available in English) | Weak | Psychoeducation Outcomes Questionnaire  (POQ) Patients acknowledged: (1) the importance of delivered information; (2) an opportunity to share their experience with the illness with others during the treatment group sessions; and (3) better reconciliation with the fact of being ill. They welcomed participation of their relatives in the programme.  The relatives acknowledged: (1) the importance of delivered information; (2) acceptance that medication was necessary; (3) increased trust in psychiatry;(4) acquired skills on how to behave towards the ill; (5) knowledge that the problem behaviour is not always deliberate; (6) acceptance of the biological origins of the illness; and (7) the feeling of not being alone. |  |
| 14.India  (Balaji, 2012) | 1.Collaborative community based care:  Psycho-education  Adherence management  Rehabilitation  Referral to community agents | Community Lay Health Workers | Supervised by mental health specialist e.g. psychiatric social worker.  Psychiatrist provide clinical leadership  CLHW received training to act as positive role  models in their interactions with the family. | In-depth interviews 32 patients, 38 caregivers | Structured interviews | Qual | Effectiveness not addressed in this developmental work | Qual - thematic | Adequate | Some caregivers reported concern that home visits would lead to their family member’s illness being disclosed leading to gossip and ridicule in the community. One participant was only willing to accept the intervention if it was not delivered in their home Training of health workers included strategies for minimising risk of disclosure.  24 of 67 families refused the intervention as they were ‘not interested’ or thought it would not be helpful. Fears that home visits were attempts at religious conversion to Christianity.  Some participants expressed a preference for female health workers  Participants expected health workers to be educated and knowledgeable on the illness  Overall, intervention components relevant and important for participants  Participation -  Engaging caregivers was not feasible in 25% of cases as they were employed or could not be present for other reasons  Targeted number of sessions could not be met if caregivers not available for visits or when patients symptomatic | Patients and Caregivers emphasized the need for home visits to be scheduled at convenient times.  Some caregivers concerned about other commitments  Out of 43 people who consented, only 30 received the intervention, the others were not contactable, or had been admitted  Intervention materials could not be used with 5 participants who could not read. Verbal explanations were acceptable and feasible in all cases  In one case, referral to community agents was not feasible as the participant could not afford to travel there  Health workers found social skills training difficult and requested more training. They found supervision sessions helpful.  There were some feasibility  barriers. Example: content on health promotion on healthy diets was not feasible for some participants |
| 15. Brazil  (Zimmer, 2006) | 1.CBT – subprograms – cognitive differentiation, social perception, verbal communication, social skills, interpersonal problem solving  Variety of tools and materials used for different sub programmes | Not reported | One of the authors (M. Zimmer) was directly trained by developer of the programme | 22 patients | Individual expressions of positive and negative perceptions around the exercises involved in the intervention  (written and verbal accounts) | Qual | Effectiveness not reported in this qual study. However authors note in introduction that there is substantial evidence from controlled studies, that indicate the benefits of  IPT in the improvement of cognitive and social performance and in the reduction in severity of  some schizophrenia symptoms. | Content analysis of group discussions with an a priori framework based on discussions of professional teams implementing the intervention | Strong | The exercises of cognitive differentiation and verbal communication are experienced as  repetitive and monotonous, as can be seen by the patients’ statements: “I don’t like the exercises  with cards, they are always repetitive;” “I think there’s no use in separating cards, it’s very boring;”  Most patients questioned cognitive differentiation and verbal communication exercises because they could not find any use for them in their daily life. “What’s the use of these lists?;” “I think this is useless, we're just pretending.”  Patients have great difficulty in participating in more theoretical activities  Patients prefer the practical exercises, such as those of the social perception, social skills and, more specifically, interpersonal problem solving subprograms.  Positive aspects noted for exercises of social perception, social skills and problem solving “This activity makes us think;” “My mother says I’m less anxious, I don’t keep walking back and forth;”  Satisfaction with psychoeducation “I was relieved to find out that other people also feel what I feel;”  Difficulties with cognitive differentiation and verbal communication presented as justifications for the low motivation and participation of patients in training groups. | Participants had difficulty in performing exercises that required writing sentences or taking  instruction notes for home activities. This can be exemplified by the following statement: “I don’t  like writing, my handwriting is not good;”  Although there were no illiterate participants,  the lower the schooling level, the more difficult it was to motivate them for verbal communication tasks (anecdotal/meeting reports) |
| 16. South Africa, (Pooe, 2010) | 1.Adapted ‘Alliance Programme’ Psycho-education material  (simplified and illustrated version of the original Alliance Programme)  3 one hour sessions over 3 weeks | Qualified mental health professionals | Not reported | 9 in-patients  9 out-patients in initial sample  Study did not disaggregate in analysis  15 patients completed the study | Semi–structured interviews  Two focus groups:  Group A- original version of the Alliance  Programme.  Group B –adapted version of the Alliance Programme. | Qual | Data on effectiveness not included in this qualitative study | Inductive  qualitative content analysis | Strong | Group A participants seemed bored and uncomfortable; there was very little interaction between the participants. The facilitator did most of the talking, a top-down form of interaction  Group B was more engaging than the  Group A. The interaction was spontaneous amongst  group participants and with the facilitator  Group A’s feedback on the original version was that it was difficult; the language used was too technical. They believed that lay people and not just  patients with schizophrenia would struggle to understand the programme.  Group B participants found the adapted version easy to read and understand. They particularly liked the fact that even their relatives could understand it. They claimed to understand their mental illness better due to the examples given in the booklet. | Due to the different dialects of Setswana spoken around Tshwane, the situation necessitated an interpreter to allow for a uniform language. This however proved to be an expensive exercise as a lot of time was wasted. This will be difficult to replicate in natural settings. |
| 17. South Africa  (Asmal 2013) | 1.Four multi-family groups of adult outpatients with schizophrenia and their caregivers. Six sessions per group | Psychiatric nurse | Nurse with 20 years  community psychiatry experience and additional training in qualitative methods | 20 patients and 20 family member | Semi-structured interviews | Qual | Effectiveness data not reported - qualitative study | Thematic analysis | Strong | Overall adherence to the sessions was 75.0% (79.5% among relatives; 70.5% among patients). Attendance rates  remained stable without a drop-off as the sessions progressed.  Several measures were implemented to enhance adherence: sessions were arranged to coincide with scheduled clinic treatment , the study coordinator  telephoned a reminder to relatives the day before each session; relatives and patients were modestly reimbursed  for transport costs.  Patients and relatives agreed that a psycho-educational frame helped to address gaps in their knowledge about the biology of schizophrenia and its treatment. Participants found the content of each session relevant and accessible. There were differences in interest between patients and relatives in other topics of the programme.  Patients, for example, placed more emphasis on physical and verbal abuse within communities, loneliness and difficulty abstaining from illicit substances. Relatives, however, emphasized hostile behaviour displayed by the patient especially when using substances, poverty, physical illness, lack of support from other family members and community violence as major stressors of being a carer.  Participants did not express concern about speaking in a group setting and no potential participant declined to join the study because of the multi-family format. Relatives felt that the opportunity to share experiences with others who faced similar challenges was valuable and helped to decrease the sense of isolation. On the other hand, some relatives thought that it would be beneficial to have sessions that did not include the patient with schizophrenia. |  |
