# Supplementary material for: Systematic review of feasibility and acceptability of psychosocial interventions for schizophrenia in low and middle income countries
Source: BMC Psychiatry. 2015 Feb 12;15:19. doi: 10.1186/s12888-015-0400-6 (PMC4382830; doi:10.1186/s12888-015-0400-6)
Supplement: Additional file 3: — Quality assessment. [file 12888_2015_400_MOESM3_ESM.docx]

**Appendix 3 Quality Assessment Checklists**

1. **Quantitative table checklist ( 1 = STRONG, 2 = MODERATE, 3 = WEAK, 4 = UNKNOWN)**

| **Article** | **Clear aims stated** | **Selection bias addressed** | **Appropriate study design** | **Confounders addressed** | **Blinding** | **Valid and reliable tools** | **Withdrawals and dropouts addressed** | **Intervention integrity** | **Appropriate statistical methods** | **Value of research discussed** | **Assessment** |
| --- | --- | --- | --- | --- | --- | --- | --- | --- | --- | --- | --- |
| China  (Xiong, 1994) | 2 | 1 | 2 | 1 | 1 | 1 | 2 | 4 | 1 | 3 | 2 Moderate |
| China  (Ran, 2002) | 1 | 1 | 1 | 1 | 2 | 2 | 1 | 2 | 1 | 1 | 1 Strong |
| China  (Zhang, 1994) | 1 | 1 | 2 | 1 | 4 | 1 | 4 | 3 | 2 | 2 | 2 Moderate |
| China  (Zhang, 1993) | 1 | 2 | 2 | 3 | 3 | 2 | 2 | 2 | 2 | 2 | 2 Moderate |
| Turkey  (Tas, 2012) | 1 | 2 | 1 | 1 | 1 | 2 | 1 | 1 | 1 | 1 | 1 Strong |
| Poland  (Slupczynka, 1999) | 1 | 4 | 3 | 3 | 2 | 2 | 1 | 2 | 1 | 1 | 2 Moderate |
| Poland  (Chadzynska, 2011) | 1 | n/a | 1 | n/a | 2 | 3 | n/a | n/a | 2 | 2 | 2 Moderate (survey not intervention) |
| Egypt  (Gohar, 2013) | 1 | 2 | 2 | 1 | 2 | 3 | 3 | 2 | 1 | 1 | 2 Moderate |
| Chile  (Cacqueo-Urizar, 2009) | 1 | 1 | 1 | 1 | 2 | 2 | na | na | 1 | 1 | 1 Strong |
| India  (Kulhara, 2009) | 1 | 1 | 1 | 1 | 1 | 1 | 2 | 1 | 1 | 1 | 1 Strong |
| Brazil  (Cabral, 2009) | 1 | 1 | 2 | na | na | 2 | 1 | na | 1 (only percentages?) | 2 | 1 Strong |
| Thailand  (Worakul, 2007) | 1 | 4 | 3 | 3 | 4 | 4 | 4 | 4 | 2 | 3 | 4 Unknown |
| Czech Republic  (Motlova 2006) | 1 | 2 | 1 | 4 | 4 | 4 | 4 | 2 | 1 | 1 | 3 Weak |

1. **Qualitative data study checklist (Y=Yes, N=No, I=Insufficient information) (STRONG, ADEQUATE, WEAK, UNKNOWN)**

| **Article(s)** | **Clear aims** | **Appropriate methodology** | **Appropriate research design** | **Detailed, justified, recruitment strategy** | **Appropriate data collection methods** | **Researcher-participant relationship considered** | **Ethical issues considered** | **Rigorous data analysis** | **Clear findings** | **Value of research discussed** | **Assessment** |
| --- | --- | --- | --- | --- | --- | --- | --- | --- | --- | --- | --- |
| India  (Balaji, 2012) | Y | Y | Y | I | I | N | Y | N | Y | Y | Adequate |
| Brazil  (Zimmer, 2006) | Y | Y | Y | Y | Y | N | I | Y | Y | Y | Strong |
| South Africa  (Pooe, 2010) | Y | Y | Y | I | Y | Y | Y | I | Y | N | Strong |
| South Africa  (Asmal, 2013) | Y | Y | Y | Y | Y | N | Y | I | Y | Y | Strong |
